# Supplementary material for: Uridine phosphorylase-1 supports metastasis by altering immune and extracellular matrix landscapes
Source: EMBO Rep. 2025 Jul 23;26(17):4248–82. doi: 10.1038/s44319-025-00520-7 (PMC12420820; doi:10.1038/s44319-025-00520-7)
Supplement: Supplementary file 1 — Appendix [file 44319_2025_520_MOESM1_ESM.pdf]

APPENDIX TABLE OF CONTENTS

Appendix Figure	Page
Appendix Figure S1: Characterising circulating metabolic signatures of cancer	2
Appendix Figure S2: Plasma uracil does not correlate with clinical subtype or organ of metastasis in metastatic breast cancer patients	3
Appendix Figure S3: The metabolic characterisation of *Upp1* knockout mice	4
Appendix Figure S4: Gene expression profiles of lineage markers	5
Appendix Figure S5: *Upp2* expression profiles across immune cell lineages	6
Appendix Figure S6: *Upp1* expression in immune cells of interest	7
Appendix Figure S7: 5-Benzylacyclouridine inhibits the metabolic activity of UPP1 but does not affect primary tumour growth	8
Appendix Figure S8: No significant *Upp1* dependent changes to immune landscapes in lymph nodes and spleen	9
Appendix Figure S9: The promigratory matrix deposited by fibroblasts is dependent on exogenous uracil and not endogenous *Upp1*	10
Appendix Figure S10: Mouse weights and tumour weights are unaltered in the presence and absence of *Upp1*	11
Appendix Figure S11: Pentose phosphate is not altered in the absence of *Upp1* or in neutrophil depleted conditions	12
Appendix Figure S12: Exogenous uracil does not influence the cell surface expression of α_M integrin on neutrophils	13
Appendix Figure S13: Expression of markers of NETosis is not altered in the lungs of MMTV-*PyMT* mice in the presence or absence of *Upp1*	14
Appendix Figure S14: Neutrophil depletion for 2 – 3 weeks did not significantly alter metastasis in tumour-bearing MMTV-*PyMT* mice	15
Appendix Table S1: Serum metabolites that correlate with metastasis in the MMTV-*PyMT* model of mammary cancer	16
Appendix Table S2: Antibody panel for neutrophil characterisation	17
Appendix Table S3: Antibody panel for lung immunophenotyping	17
Appendix Table S4: UPP1 expression is not upregulated in cancer associated fibroblasts in comparison to normal fibroblasts	17
Appendix Table S5: Isotopically labelled internal standards for CD11b-DTR metabolomics	18
Appendix Table S6: Antibody panel for lymphoid characterisation	19
Appendix Table S7: Antibody panel for intracellular characterisation of T cell effector function	19
Appendix Table S8: qRT-PCR primer sequences	19

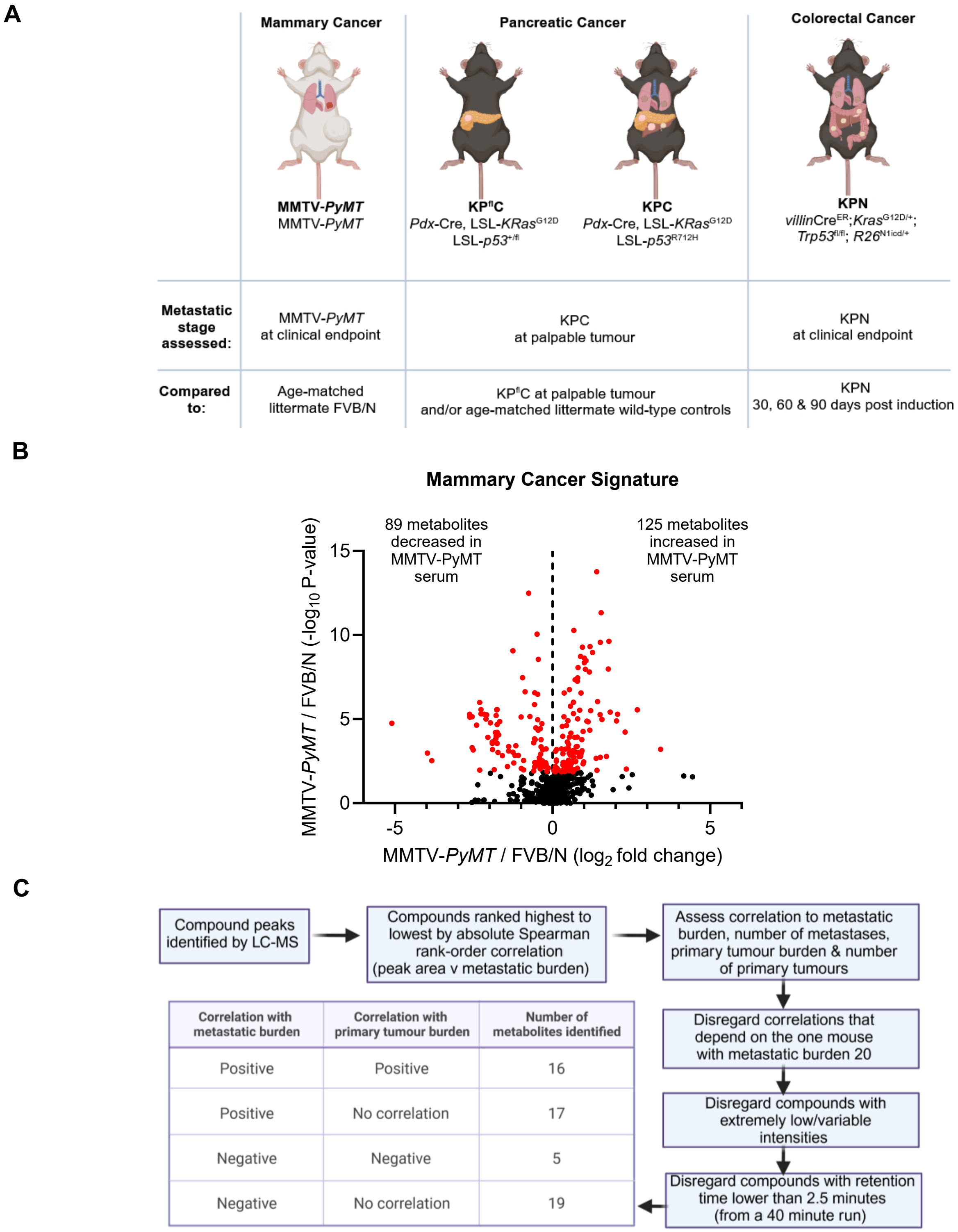

Appendix Figure S1: Characterising circulating metabolic signatures of cancer. (A) Schematic representation of genetically engineered mouse models used in Figure 1. (B) Volcano plot representing compounds identified in the serum of female MMTV-*PyMT* tumour-bearing mice (n=29 mice) and age-matched female FVB/N (n=9 mice). (C) Schematic representation of the methodology used to identify metabolites that correlate with metastasis independent of primary tumour burden. Molecular weights, retention times and compound identifications confirmed by comparison to commercial standard for each of the groups described are detailed in Appendix Table S1.

Data information: In (B), each dot represents an individual metabolite, and metabolites highlighted in red were determined as being statistically significantly different in the serum of MMTV-*PyMT* mice compared to FVB/N WT using p-values calculated with unpaired t-test and adjusted with Benjamin Hochberg false discovery rate calculation.

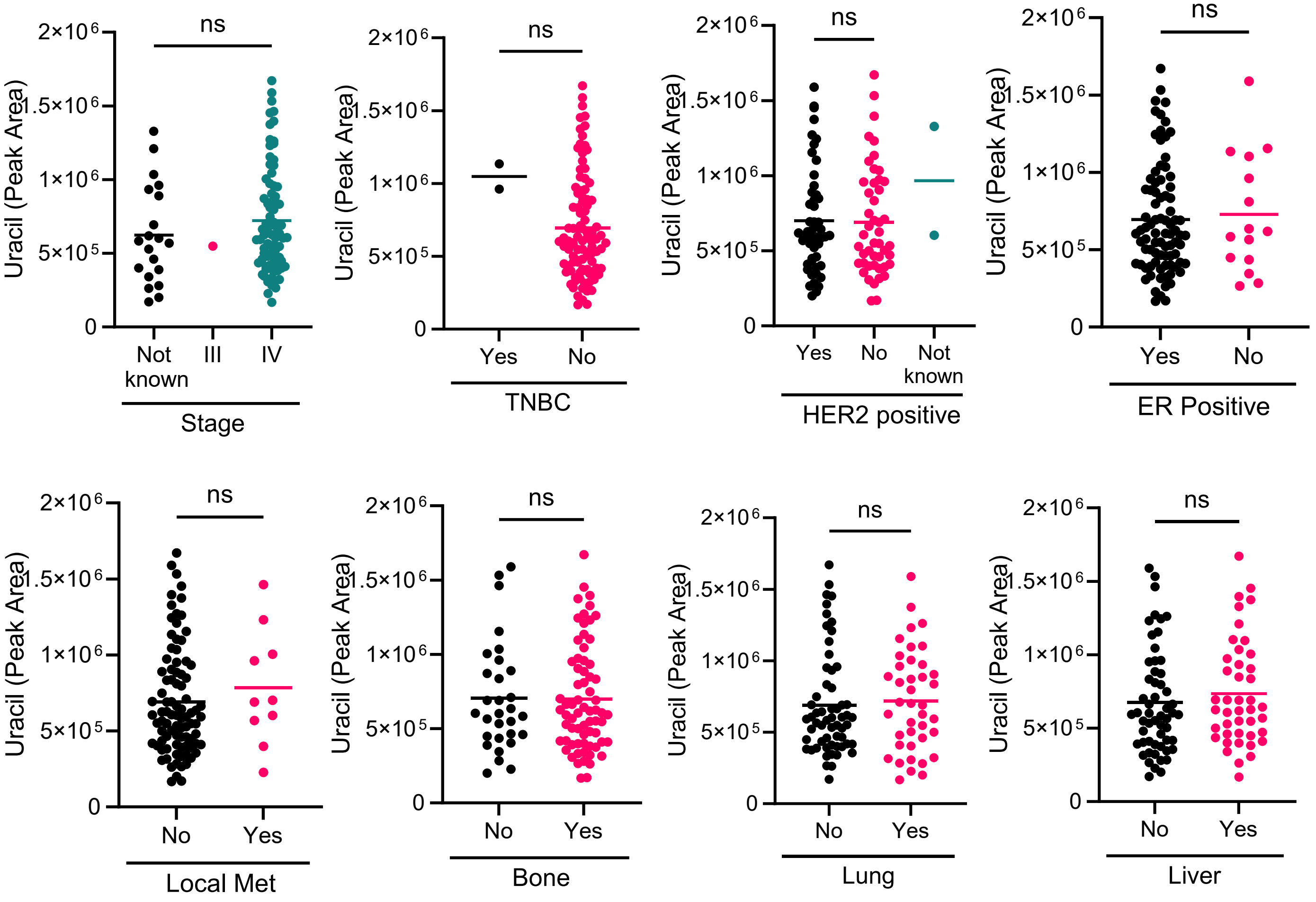

Appendix Figure S2: Plasma uracil does not correlate with clinical subtype or organ of metastasis in metastatic breast cancer patients. Plasma uracil levels were assessed against all associated clinical data regarding stage and subtype (top row), and organ of metastasis (bottom row) (n=99 patients).

Data Information: Each dot represents an individual patient, mean is represented by horizontal line, statistics calculated using unpaired t-test, ns = not-significant.

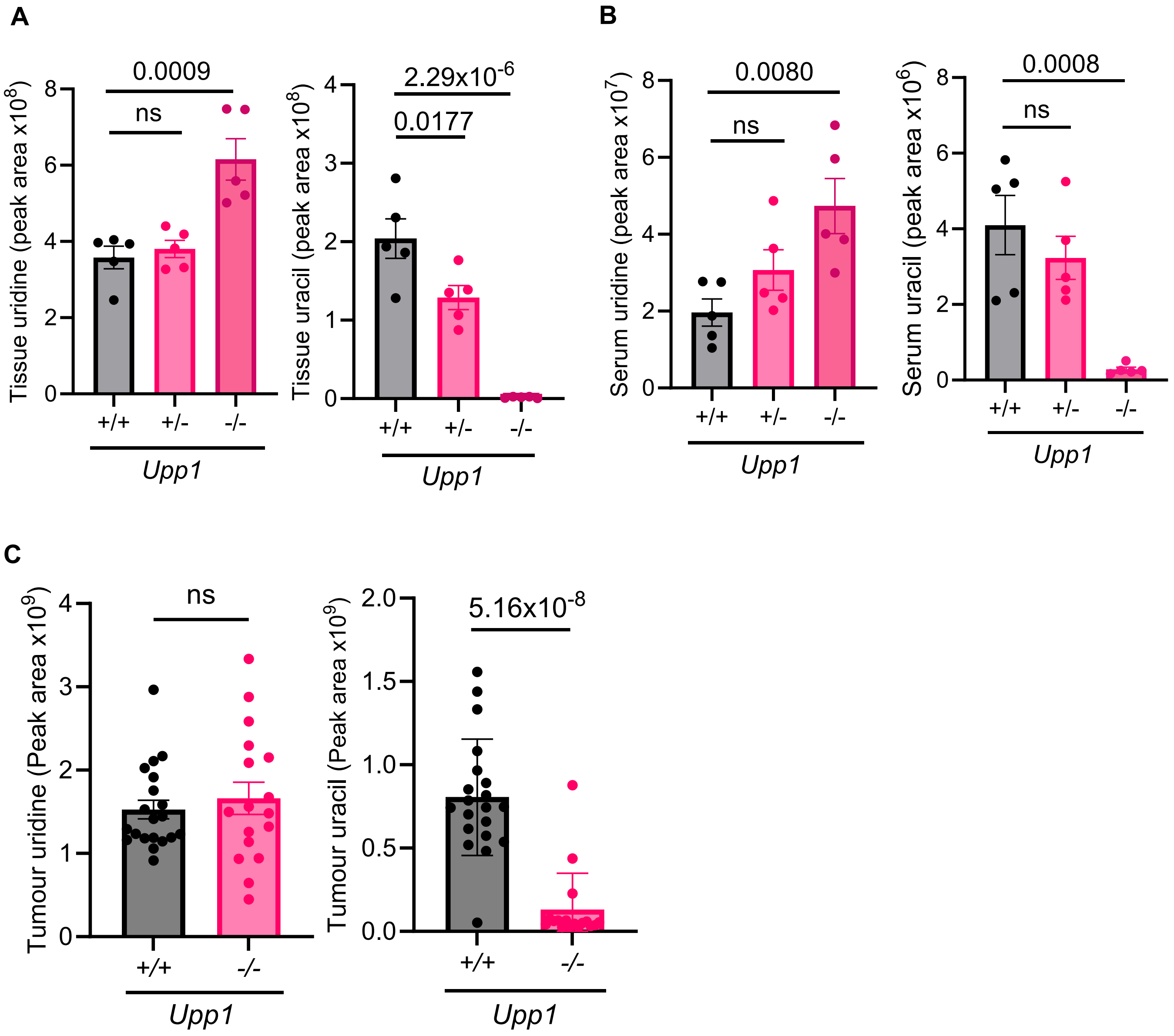

Appendix Figure S3: The metabolic characterisation of *Upp1* knockout mice. Polar metabolites were extracted from (A) tissue and (B) serum of FVB/N *Upp1*$^{+/+}$, *Upp1*$^{+/-}$, and *Upp1*$^{-/-}$ mice, and uridine and uracil levels were assessed by LC-MS. Tissue, in this instance, refers to pancreas (n=5 mice per experimental group). (C) Polar metabolites were extracted from mammary tumours of MMTV-*PyMT* *Upp1*$^{+/+}$ (n=20 mice) and *Upp1*$^{-/-}$ (n=17 mice) mice at clinical endpoint, and uridine and uracil levels were assessed by LC-MS.

Data Information: In each case, dots represent individual mice, bar graphs are mean ± SEM. In (A and B) statistics are one-way ANOVA. When one-way ANOVA produced $p<0.0001$, t and DF values provided by Šídák's multiple comparisons test were used to calculate the exact p-value. In (C) statistics are unpaired t-test, ns = not significant.

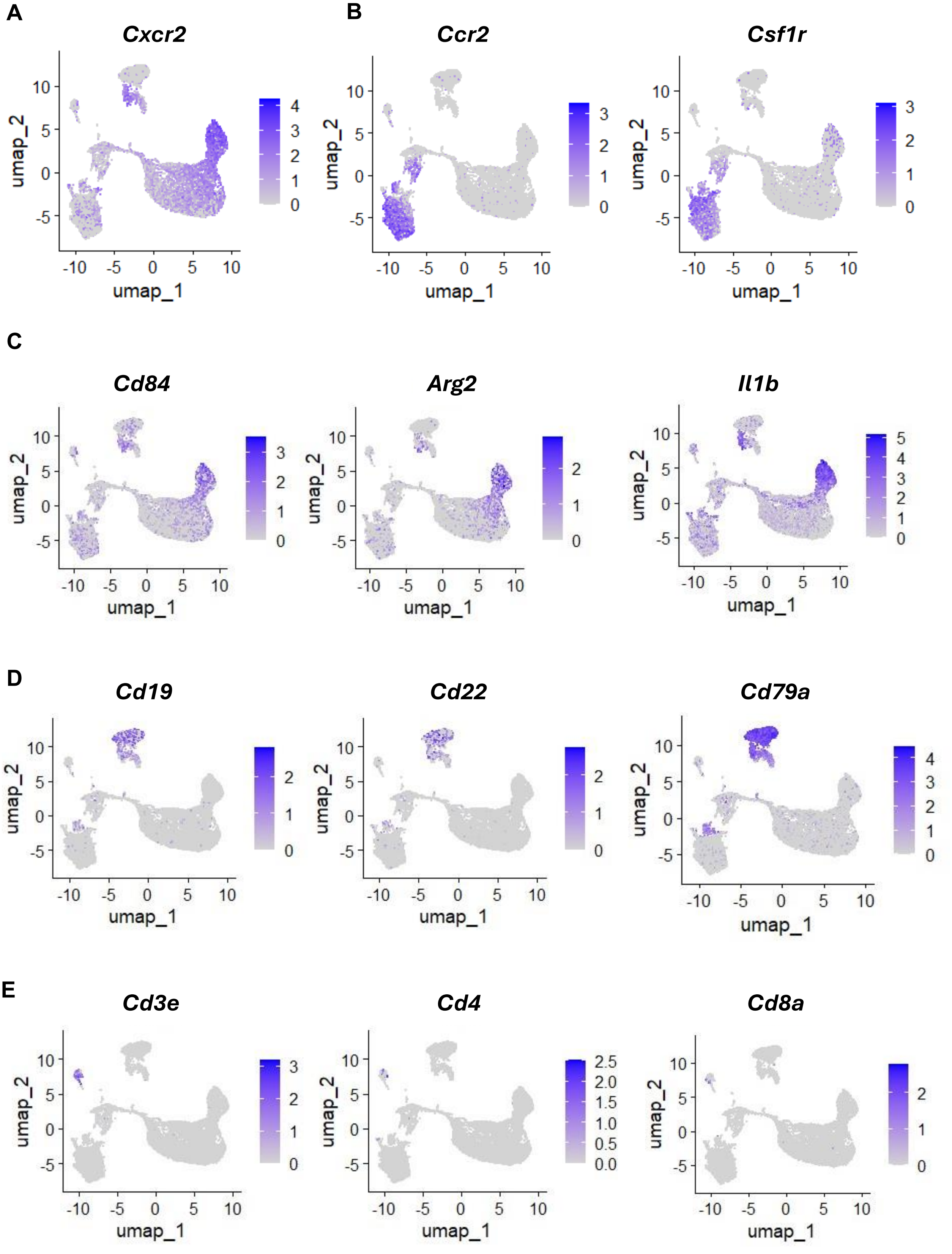

Appendix Figure S4: Gene expression profiles of lineage markers. UMAP plots from scRNA-Seq data from splenic cells isolated from MMTV-*PyMT* tumour-bearing mice (n=3 mice pooled). Increased average expression of specified genes are represented by increased colour, as a surrogate to identify (A) neutrophils, (B) monocytes, (C) granulocytic myeloid suppressor cells, (D) B cells, (E) T cells.

Dat Information: Data source is (Alshetaiwi et al, 2020). Statistics have not been performed on this data.

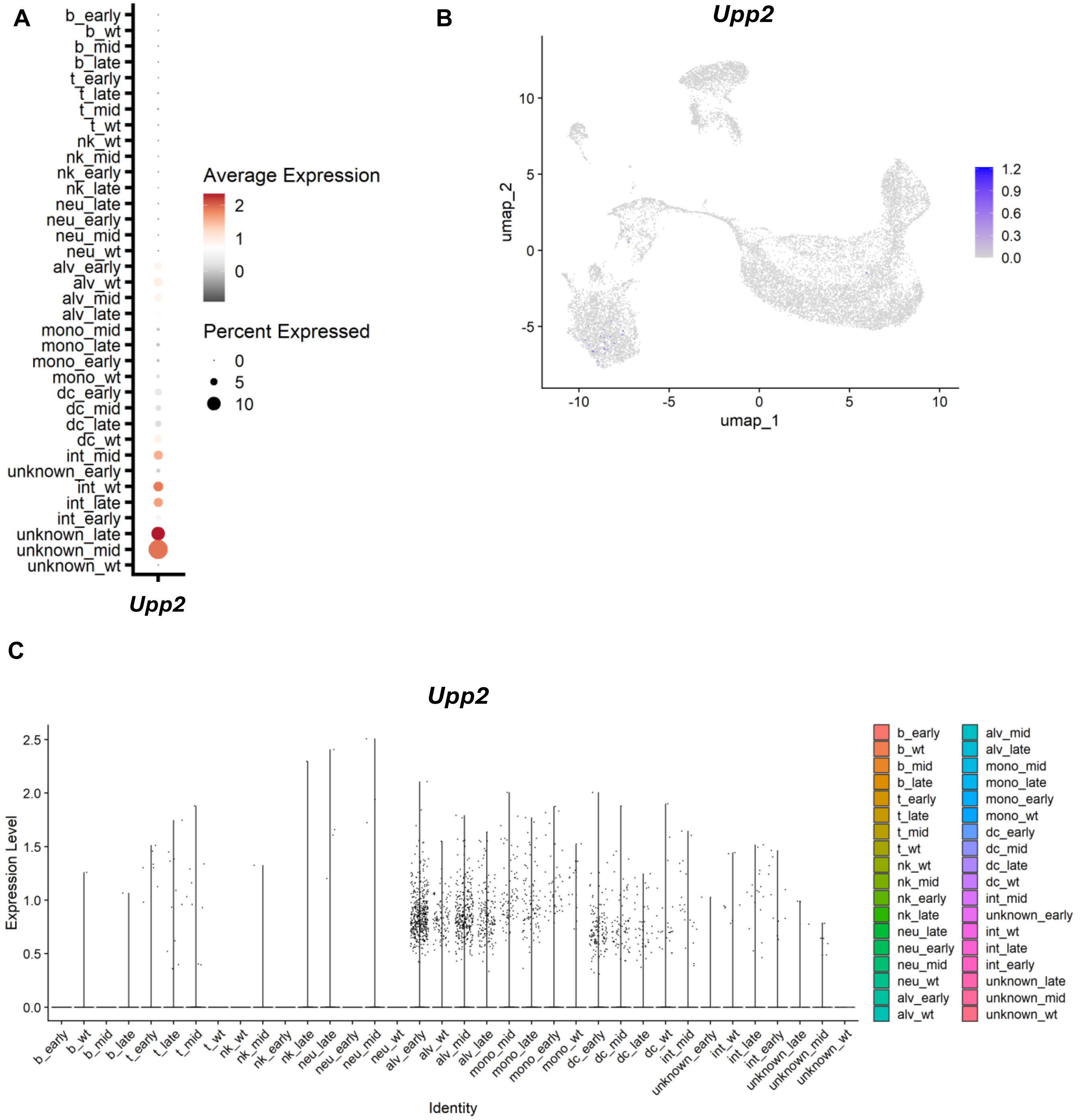

Appendix Figure S5: *Upp2* expression profiles across immune cell lineages. (A) *Upp2* data was extracted from https://github.com/chris-mcginnis-ucsf/pymt_atlas allowing *Upp2* to be assessed in immune cells from the lungs of wild-type (WT) or MMTV-*PyMT* tumour bearing mice at varying timepoints in tumour progression (as assigned by early, mid or late) (n=29 samples). Cells classified as b (B cells), t (T cells), nk (NK cells), neu (neutrophils), alv (alveolar macrophages), mono (monocytes), dc (dendritic cells), int (interstitial cells) and cells of unknown origin. Dot size represents the percentage of cells expressing *Upp2*, and dot colour represents average *Upp2* expression levels (B) UMAP plot of *Upp2* from scRNA-Seq from splenic cells isolated from MMTV-*PyMT* tumour-bearing mice (n=3 mice pooled). Increased average expression of *Upp2* is represented by increased colour. (C) *Upp2* expression in individual cell types from wild-type (WT) mice (n=5 mice pooled) in comparison to tumour-bearing MMTV-*PyMT* (n=3 mice pooled).

Data Information: In (A), data source is (McGinnis et al, 2024). In (B – C), data source is (Alshetaiwi et al, 2020). Statistics have not been performed on this data.

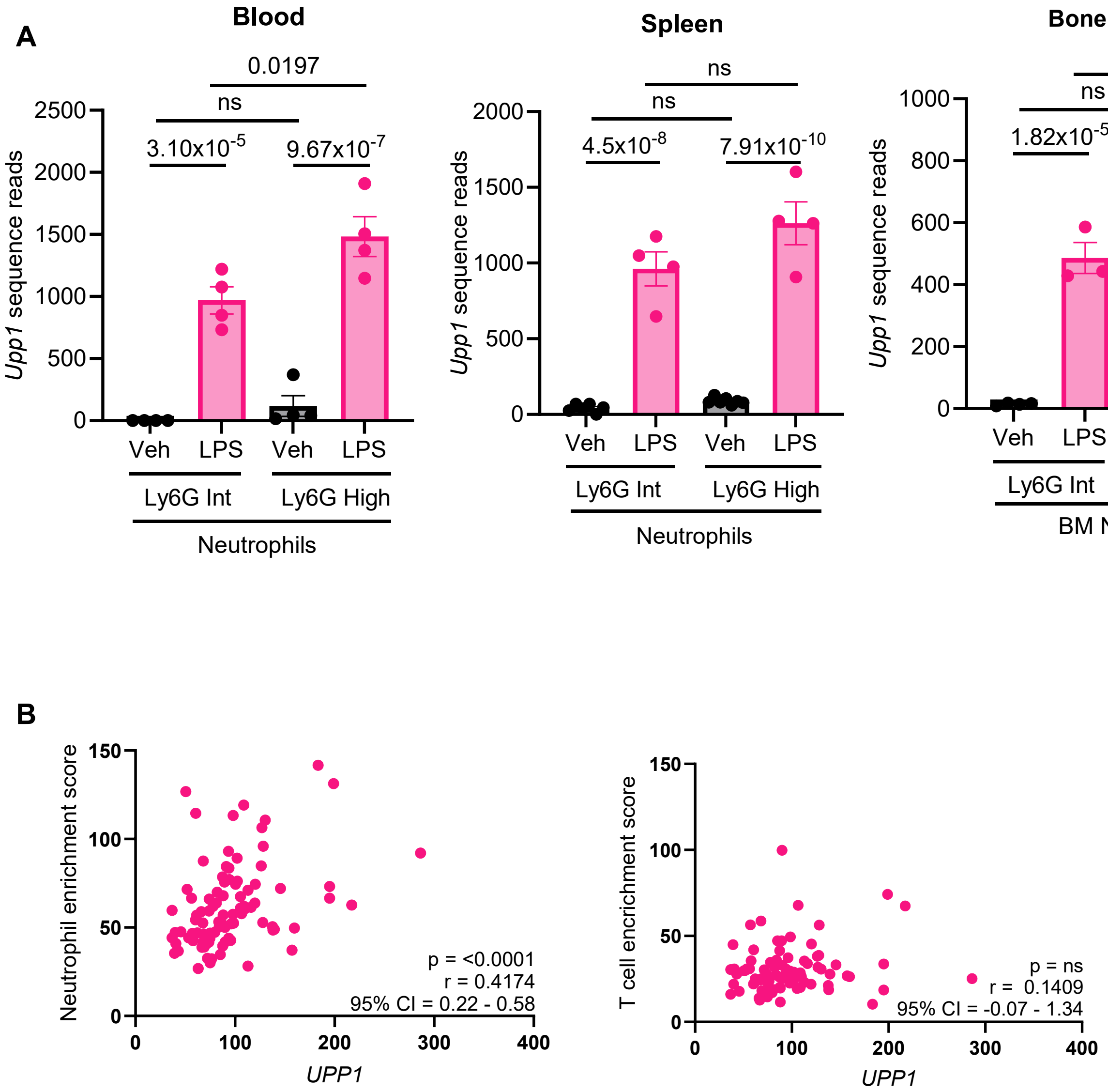

Appendix Figure S6: *Upp1* expression in immune cells of interest. (A) *Upp1* detected by RNA-Seq in $CD11b^{+}Ly6G^{+}$ neutrophils flow cytometry sorted from the blood, spleen and bone marrow of C57BL/6 mice 24 hours post IP dosing of 1mg/kg LPS. Cell surface levels of Ly6G were determined as intermediate (immature neutrophils) or high (mature neutrophils). Number of mice per experimental group: n=4 mice per group for blood, for spleen n=6 vehicle and n=4 LPS treated Ly6G intermediate sorted cells, and n=7 vehicle and n=4 LPS treated Ly6G high sorted cells, for bone marrow n=4 vehicle treated mice and n=3 LPS treated mice per group. (B) Neutrophil enrichment score, and T cell enrichment score, were determined using Microenvironment Cell Populations counter (MCP) methodology (Becht et al., 2016) on human colon cancer dataset GSE33113 (n=90 patients).

Data information: In (A), data source is (Mackey et al., 2021), dots represent individual mice, mean ± SEM, statistics are one-way ANOVA. When one-way ANOVA produced $p<0.0001$, t and DF values provided by Šídák's multiple comparisons test were used to calculate the exact p-value. In (B), data source is GSE33113 dots represent individual patients; statistics are Spearman correlation.

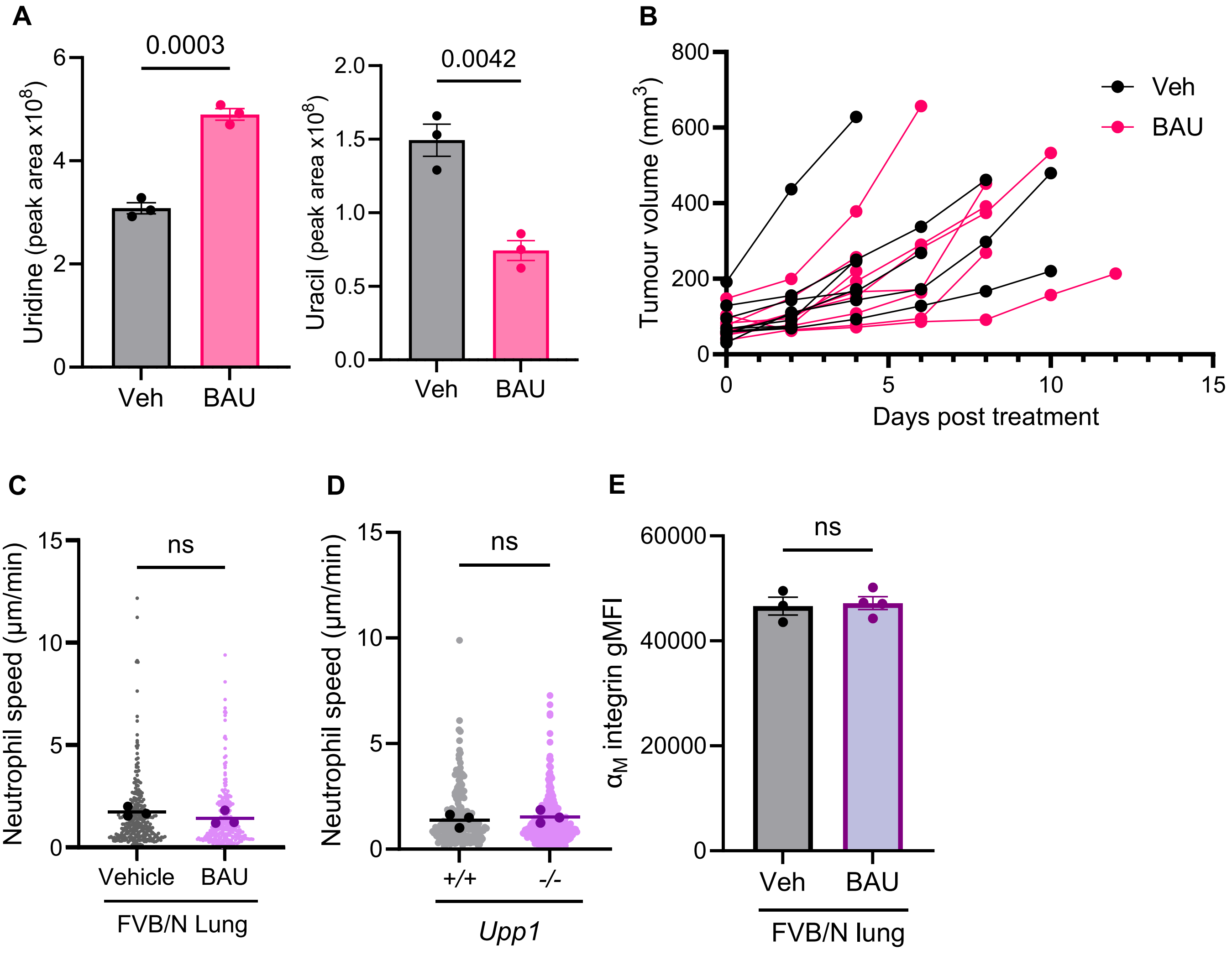

Appendix Figure S7: 5-Benzylacyclouridine inhibits the metabolic activity of UPP1 but does not affect primary tumour growth. (A) *In vivo* validation of 5-benzylacyclouridine (BAU) as an inhibitor of UPP1. C57Bl/6 mice (n=3 per experimental group) were treated for 3 days with vehicle or BAU, and uridine and uracil levels assessed by LC-MS. Tissue presented is from pancreas. (B) Tumour volumes calculated from female mice transplanted orthotopically with tumour fragments obtained from KP mice. Mice were dosed with vehicle (n=10 mice) or BAU (n=9 mice) following detection of measurable tumours. Tumour measurements were determined 3 times per week by calliper measurement. (C) Neutrophil speed was assessed in the lungs of FVB/N mice by live cell imaging of precision cut lung slices, comparing mice treated with vehicle or BAU for 12 days (n=3 mice per group). (D) As (C) but with assessment of neutrophil speed in the lungs of *Upp1*$^{+/+}$ or *Upp1*$^{-/-}$ mice (n=3 mice per group). (E) Cell surface levels of α_M integrin on neutrophils (CD45$^+$Ly6G$^+$CD11b$^+$) treated with BAU, as assessed by flow cytometry (n=3 vehicle-treated mice, n=4 BAU treated-treated mice).

Data Information: In (A and E), each dot is an individual mouse, bar graph is mean ± SEM, and statistics are unpaired t-test. In (B), each adjoined line represents an individual mouse, with dots representing individual measurements, no statistics have been performed. In (C and D), the lighter colour background dots represent the speed of each individual neutrophil assessed, whilst the darker forefront dots are the average neutrophil speed for each mouse. Horizonal line represents mean speed from the average of n=3 mice, and an unpaired t-test has been performed on the average speed per experiment.

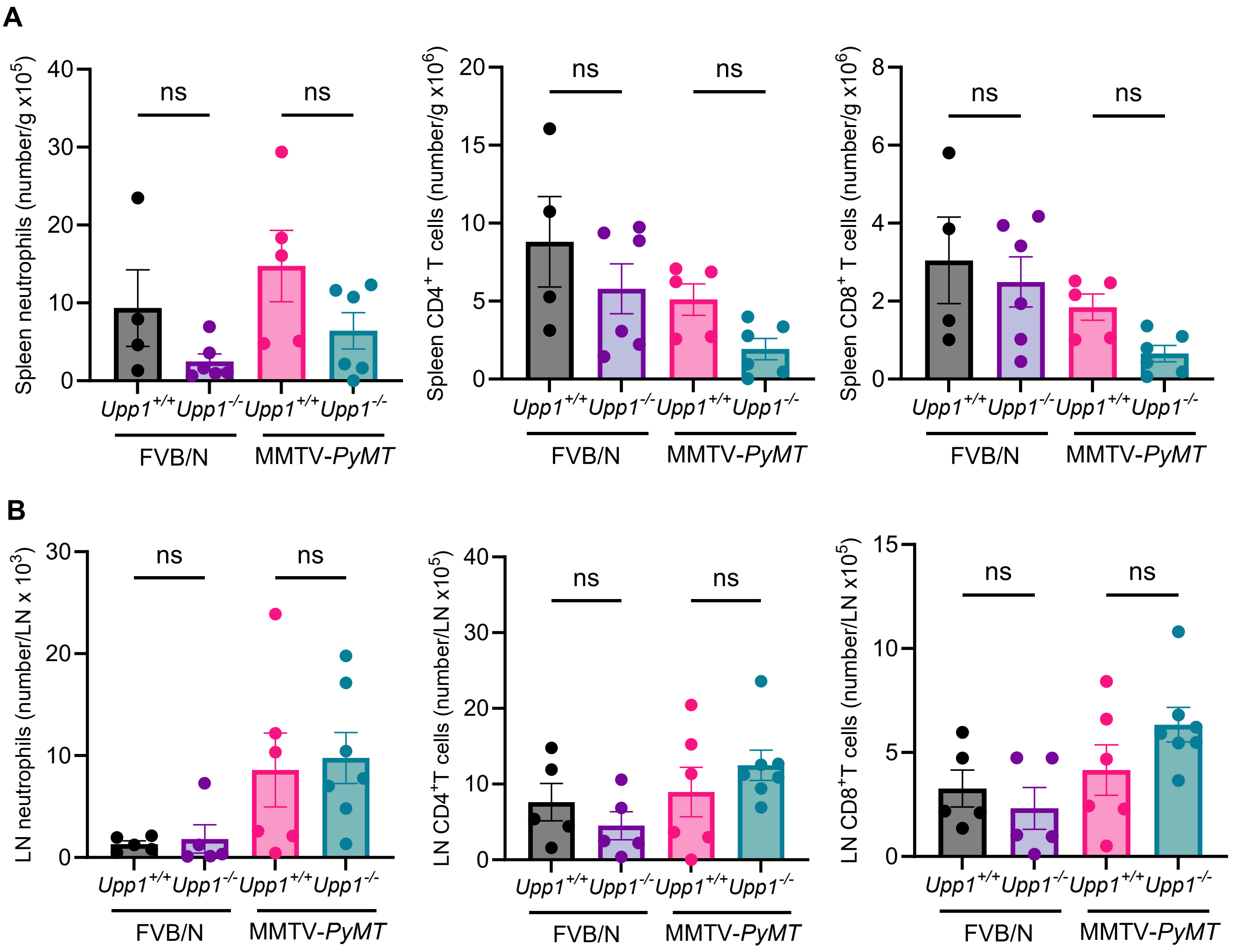

Appendix Figure S8: No significant *Upp1* dependent changes to immune landscapes in lymph nodes and spleen. Cell number per gram of (A) spleen and (B) lymph nodes (LN) for neutrophils, CD4+ T cells and CD8+ T cells, assessed by flow cytometry of *Upp1*$^{+/+}$ and *Upp1*$^{-/-}$ mice, with MMTV-*PyMT* mice bearing mammary tumours 10 – 15mm in diameter (For spleen, n = 4 *Upp1*$^{+/+}$ and n=6 *Upp1*$^{-/-}$ FVB/N mice, n=5 *Upp1*$^{+/+}$ and n=6 *Upp1*$^{-/-}$ MMTV-*PyMT* mice. For LN, n = 5 *Upp1*$^{+/+}$ and n=5 *Upp1*$^{-/-}$ FVB/N mice, n=6 *Upp1*$^{+/+}$ and n=7 *Upp1*$^{-/-}$ MMTV-*PyMT* mice).

Data Information: Dots are individual mice, graphs are mean ± SEM, statistics are one-way ANOVA, mean, ns = not significant.

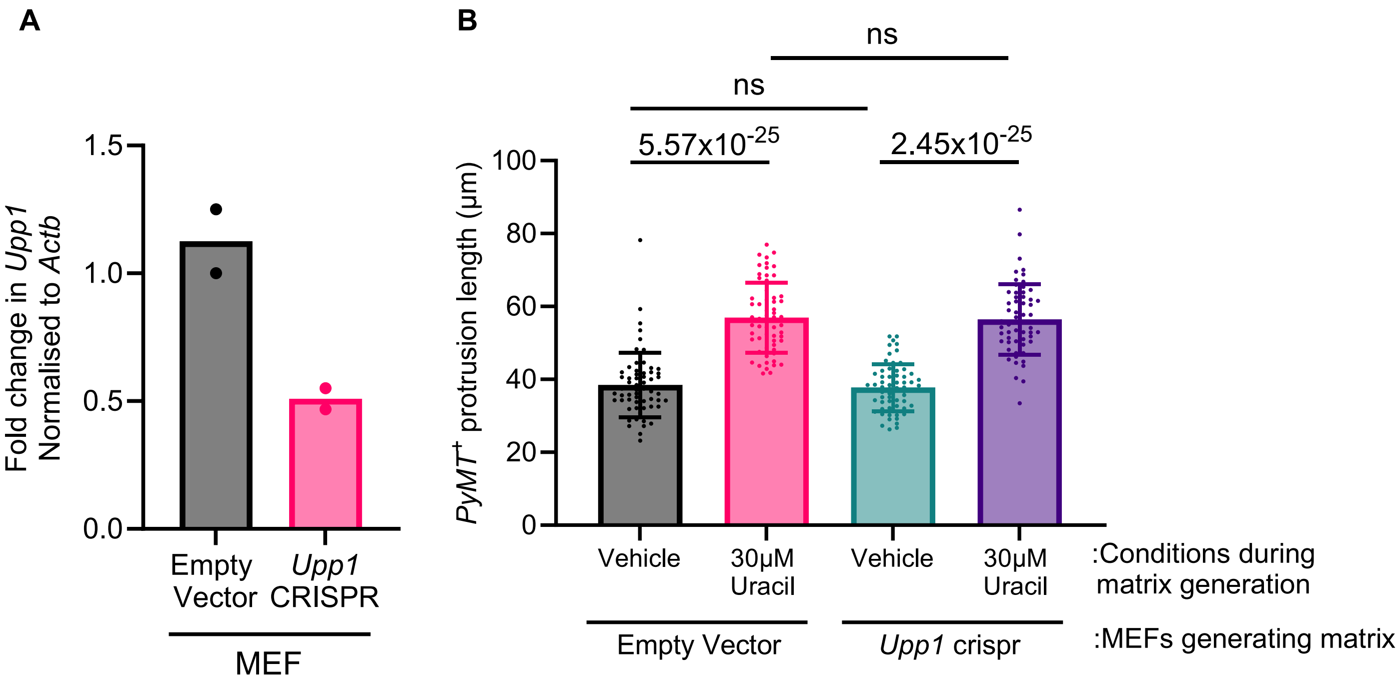

Appendix Figure S9: The promigratory matrix deposited by fibroblasts is dependent on exogenous uracil and not endogenous *Upp1.* (A) Knockdown of *Upp1* in mouse embryonic fibroblasts (MEFs) was performed using CRISPR technology. Efficiency of knockdown was assessed by qPCR, n=1 biological replicate, dots represent technical duplicates. (B) Cellular derived matrices were deposited by fibroblasts expressing empty vector, or *Upp1* CRISPR construct, in the presence and absence of 30µM uracil. After 7 days deposition, fibroblasts were removed, and the ability of the deposited matrix to support invasive *PyMT*+ cancer cell behaviour was assessed by timelapse microscopy. Dots represent the protrusion length of individual *PyMT*+ cells (n=30 cells measured per experiment, for two independent matrix depositions (n=60 cells total)).

Data Information: In (A), bar graph represents mean of technical duplicates, error bars and statistics not performed as n<3. In (B), dots represent individual cells, bar graph is mean ± SD, statistics are one-way ANOVA. When one-way ANOVA produced p<0.0001, t and DF values provided by Šídák's multiple comparisons test were used to calculate the exact p-value, ns = not significant.

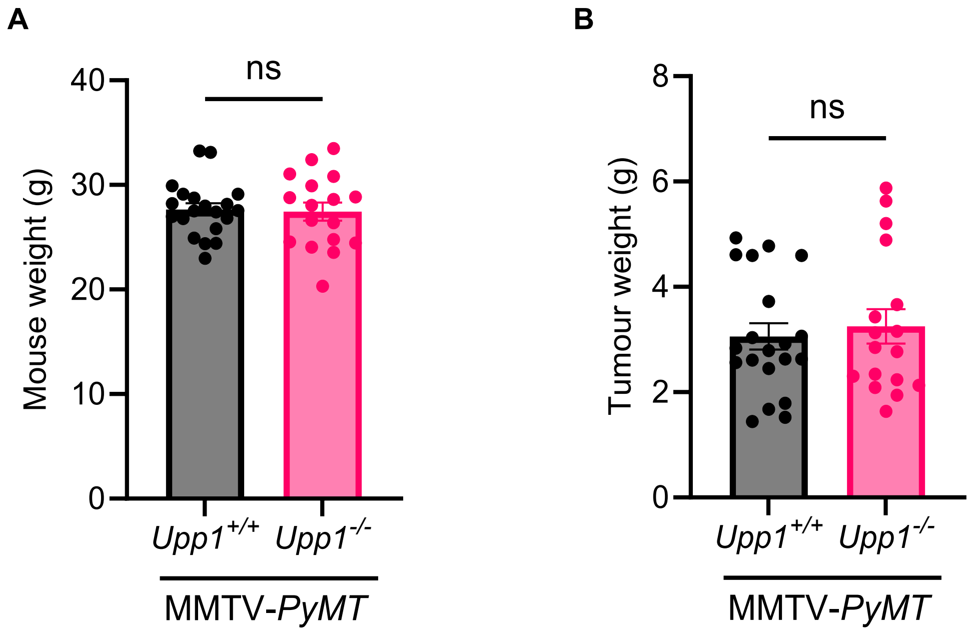

Appendix Figure S10: Mouse and tumour weights are unaltered in the presence and absence of *Upp1*. (A) Total mouse weight, and (B) weight of dissected primary mammary tumours, was assessed at clinical endpoint in MMTV-*PyMT Upp1*$^{+/+}$ (n=20 mice) and MMTV-*PyMT Upp1*$^{-/-}$ mice (n=17 mice).

Data Information: Dots represent individual mice, bar graphs are mean ± SEM, statistics are unpaired t-test, ns = not significant.

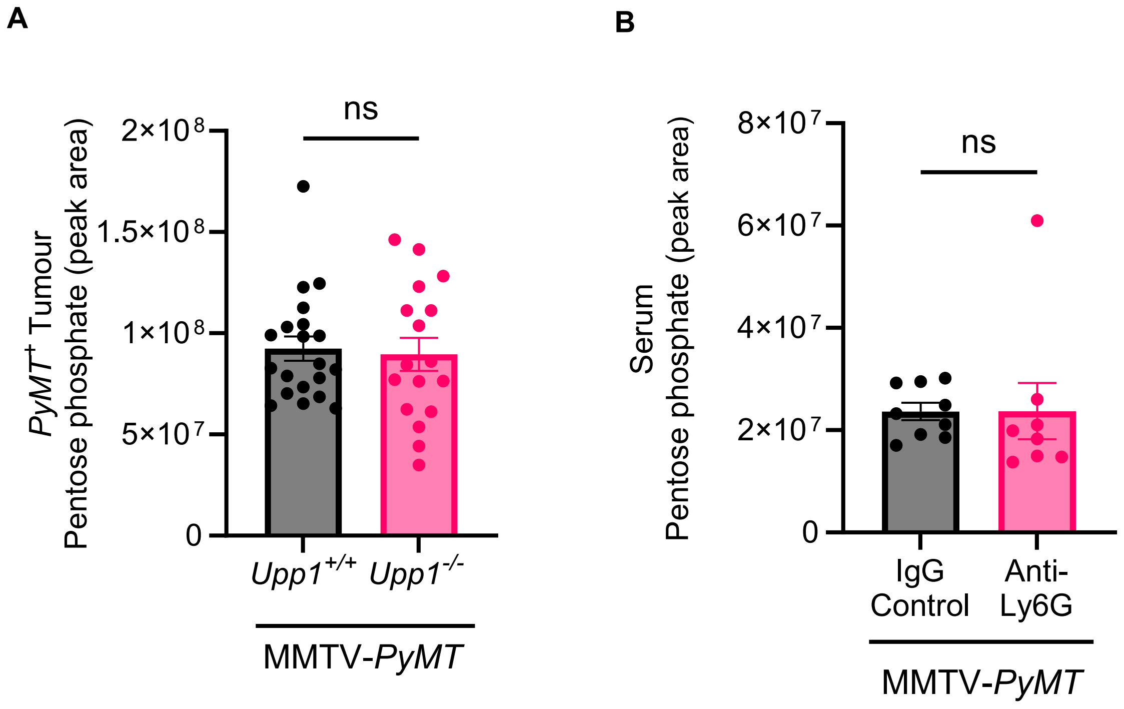

Appendix Figure S11: Pentose phosphate are not altered in the absence of *Upp1* or in neutrophil depleted conditions*.* (A) Pentose phosphate was assessed by LC-MS in mammary tumours from MMTV-*PyMT* mice, *Upp1*$^{+/+}$ (n=20 mice), *Upp1*$^{-/-}$ (n=17 mice). (B) Pentose phosphate was assessed by LC-MS in serum of MMTV-*PyMT* mice treated with IgG control (n=9 mice) or anti-Ly6G to deplete neutrophils (n=8 mice).

Data Information: Each dot represents an individual mouse, bar graph is mean ± SEM, statistics are unpaired t-test, ns = not significant.

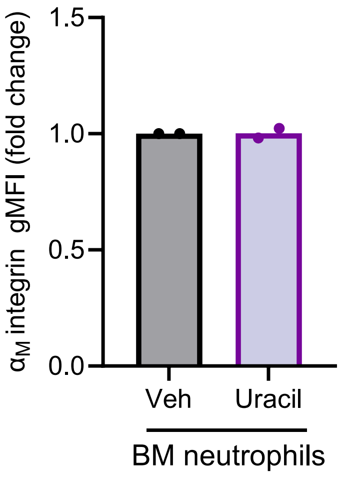

Appendix Figure S12: Exogenous uracil does not influence the cell surface expression of α_M integrin on neutrophils. Neutrophils were isolated from the bone marrow (BM) of mice, treated with 30μM uracil for 24 hours at 37°C/5%CO_2 and levels of surface α_M integrin assessed by flow cytometry (preparations from n=2 mice).

Data Information: Dots represent mean surface expression levels per individual mouse. As n=2, data are shown as points without error bars and statistics.

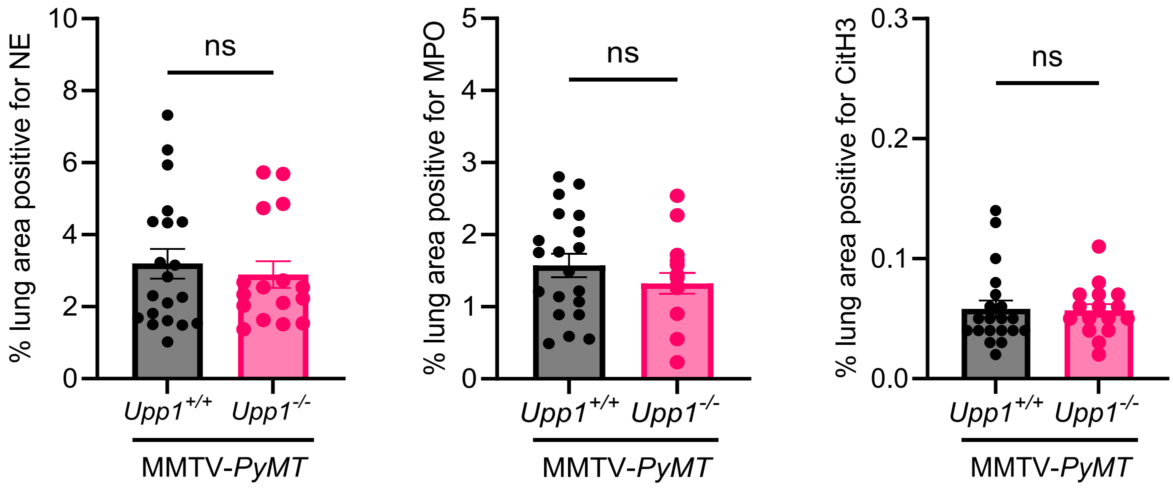

Appendix Figure S13: Expression of markers of NETosis is not altered in the lungs of MMTV-*PyMT* mice in the presence or absence of *Upp1*. Expression of neutrophil elastase (NE), myeloperoxidase (MPO), and citrullinated Histone H3 (CitH3) were assessed by immunohistochemistry in the lungs of MMTV-*PyMT* tumour-bearing mice (n=20 *Upp1*$^{+/+}$ and n=16 *Upp1*$^{-/-}$ mice). The proportion of lung area positive for each stain of interest was quantified using HALO software.

Data Information: Each dot represents an individual mouse, bar graph is mean ± SEM, statistics are unpaired t-test, ns = not significant.

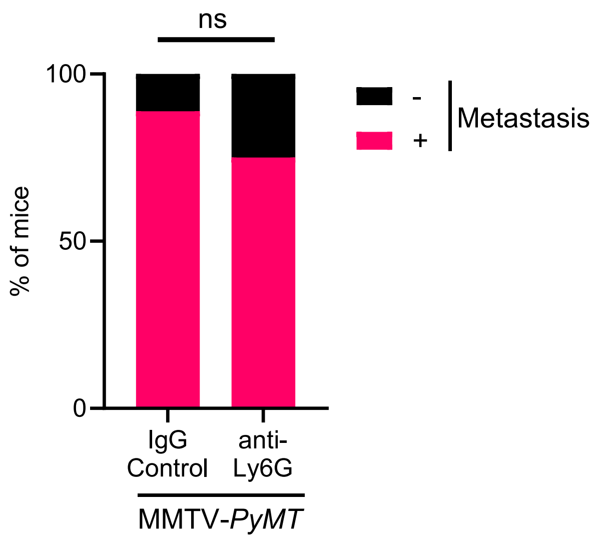

Appendix Figure S14: Neutrophil depletion for 2 – 3 weeks did not significantly alter metastasis in tumour-bearing MMTV-*PyMT* mice. Mice were treated with IgG control (n=9 mice) or anti-Ly6G (n=8 mice) once tumours reached 5mm in diameter. Mice were culled once one tumour reached 15mm diameter. Metastasis was quantified by histological examination of serial sections of FFPE lung.

Data Information: Statistics are chi-squared analysis.

Appendix Table S1: Serum metabolites that correlate with metastasis in the MMTV-*PyMT* model of mammary cancer. Spearman Correlation statistics are presented: Spearman r, 95% confidence interval, individual p value non-FDR corrected. Confirmed compound identifications are those that were validated against a commercial standard.

Spearman rank correlation (r) Compound Peak Area v Metastatic Burden	95% Confidence interval	P Value	Correlation with metastatic burden	Correlation with primary tumour burden	Molecular Weight	Retention Time (minutes)	Confirmed Compound Identification
**0.6138**	0.3088 to 0.8043	0.0004	Positive	Positive	225.07	6.97	
**0.5852**	0.2679 to 0.7880	0.0009	Positive	Positive	145.07	10.50	
**0.5827**	0.2644 to 0.7866	0.0009	Positive	Positive	201.11	8.24	
**0.5359**	0.1999 to 0.7591	0.0027	Positive	Positive	285.10	5.50	N4-Acetylcytidine
**0.5263**	0.1870 to 0.7534	0.0034	Positive	Positive	114.04	8.10	
**0.5157**	0.1729 to 0.7470	0.0042	Positive	Positive	153.05	5.50	
**0.5085**	0.1635 to 0.7427	0.0049	Positive	Positive	515.29	2.93	
**0.4121**	0.04241 to 0.6826	0.0263	Positive	Positive	132.05	9.05	3-Ureidopropionic acid
**0.4777**	0.1236 to 0.7239	0.0088	Positive	Positive	172.01	13.07	Glycerol 3-phosphate
**0.4769**	0.1227 to 0.7234	0.0089	Positive	Positive	327.12	12.45	
**0.4644**	0.1067 to 0.7156	0.0112	Positive	Positive	146.07	6.83	
**0.4429**	0.07993 to 0.7022	0.0161	Positive	Positive	189.11	13.28	
**0.4200**	0.05187 to 0.6876	0.0233	Positive	Positive	158.04	11.43	
**0.4185**	0.05008 to 0.6867	0.0239	Positive	Positive	174.10	11.19	
**0.4173**	0.04859 to 0.6859	0.0243	Positive	Positive	244.07	9.59	Pseudouridine
**0.3951**	0.02204 to 0.6715	0.0339	Positive	Positive	532.32	2.92	
**0.4254**	0.05846 to 0.6911	0.0214	Positive	No correlation	208.05	9.61	
**0.5509**	0.2203 to 0.7680	0.002	Positive	No correlation	352.08	13.05	
**0.5307**	0.1929 to 0.7560	0.0031	Positive	No correlation	299.19	5.23	
**0.5001**	0.1525 to 0.7376	0.0057	Positive	No correlation	210.04	12.21	
**0.4851**	0.1331 to 0.7284	0.0077	Positive	No correlation	130.03	12.26	
**0.4841**	0.1318 to 0.7278	0.0078	Positive	No correlation	266.05	9.65	
**0.4806**	0.1274 to 0.7257	0.0083	Positive	No correlation	326.22	2.63	
**0.4651**	0.1076 to 0.7161	0.011	Positive	No correlation	279.08	12.13	
**0.4604**	0.1017 to 0.7132	0.012	Positive	No correlation	246.09	7.08	
**0.4584**	0.09927 to 0.7119	0.0124	Positive	No correlation	113.05	3.27	
**0.4330**	0.06769 to 0.6959	0.019	Positive	No correlation	131.07	12.70	
**0.4284**	0.06206 to 0.6930	0.0204	Positive	No correlation	154.00	7.46	
**0.4178**	0.04919 to 0.6862	0.0241	Positive	No correlation	112.03	5.73	Uracil
**0.4160**	0.04710 to 0.6851	0.0248	Positive	No correlation	148.04	12.23	
**0.4111**	0.04116 to 0.6819	0.0267	Positive	No correlation	481.35	5.21	
**0.3983**	0.02585 to 0.6736	0.0324	Positive	No correlation	129.04	5.93	
**0.3857**	0.01099 to 0.6654	0.0388	Positive	No correlation	357.16	13.10	
**-0.5522**	-0.7687 to -0.2220	0.0019	Negative	Negative	146.02	13.26	Alpha-ketoglutarate
**-0.4959**	-0.7351 to -0.1471	0.0062	Negative	Negative	466.31	2.55	
**-0.5753**	-0.7823 to -0.2540	0.0011	Negative	Negative	779.55	2.76	
**-0.5605**	-0.7737 to -0.2335	0.0016	Negative	Negative	519.33	5.18	
**-0.5223**	-0.7510 to -0.1817	0.0037	Negative	Negative	102.03	13.27	
**-0.5689**	-0.7786 to -0.2451	0.0013	Negative	No correlation	607.35	3.02	
**-0.5605**	-0.7689 to -0.2223	0.0019	Negative	No correlation	541.32	3.03	
**-0.5470**	-0.7657 to -0.2149	0.0021	Negative	No correlation	236.09	5.24	
**-0.5298**	-0.7555 to -0.1918	0.0031	Negative	No correlation	134.02	14.18	
**-0.5041**	-0.7400 to -0.1577	0.0053	Negative	No correlation	246.10	5.25	
**-0.4905**	-0.7317 to -0.1401	0.0069	Negative	No correlation	548.18	12.67	
**-0.4903**	-0.7316 to -0.1397	0.0069	Negative	No correlation	384.14	12.54	
**-0.4612**	-0.7136 to -0.1027	0.0118	Negative	No correlation	304.14	13.50	
**-0.4543**	-0.7093 to -0.09403	0.0133	Negative	No correlation	246.10	3.23	
**-0.4543**	-0.7093 to -0.09403	0.0133	Negative	No correlation	868.57	2.77	
**-0.4444**	-0.7031 to -0.08176	0.0157	Negative	No correlation	148.04	13.06	
**-0.4424**	-0.7019 to -0.07932	0.0163	Negative	No correlation	545.35	5.17	
**-0.4390**	-0.6997 to -0.07506	0.0172	Negative	No correlation	369.12	12.62	
**-0.4318**	-0.6952 to -0.06628	0.0193	Negative	No correlation	567.33	5.13	
**-0.4195**	-0.6873 to -0.05127	0.0235	Negative	No correlation	549.18	12.60	
**-0.4079**	-0.6798 to -0.03732	0.0281	Negative	No correlation	275.15	15.19	
**-0.4054**	-0.6783 to -0.03437	0.0291	Negative	No correlation	261.12	10.30	
**-0.4032**	-0.6768 to -0.03172	0.0301	Negative	No correlation	132.04	12.48	
**-0.3921**	-0.6696 to -0.01854	0.0354	Negative	No correlation	175.05	12.28	N-Acetylaspartic acid

Appendix Table S2: Antibody panel for neutrophil characterisation

Target	Fluorophore	Dilution	Supplier	Catalogue Number
**Zombie Green**	Zombie Green	1/1000	Biolegend	423111
**Ter119**	FITC (Dump)	1/200	Biolegend	116206
**Nkp46**	FITC (Dump)	1/200	Biolegend	137606
**CD115 (CSF1R)**	FITC (Dump)	1/200	TONBO bioscience	35-1152-u100
**CD3**	FITC (Dump)	1/200	Biolegend	100306
**CD19**	FITC (Dump)	1/200	Biolegend	101506
**CD45**	BV650	1/200	Biolegend	103151
**CD11b / integrin α_M**	BV605	1/200	Biolegend	101257
**Ly6G**	Bv510	1/200	Biolegend	127633
**CD54**	APC	1/200	Biolegend	116120
**CD62L**	BUV395	1/200	Biolegend	740218
**CD101**	PE-Cy7	1/200	Life Tech (Invitrogen)	25-1011-82
**CD117**	Bv711	1/100	Biolegend	105835
**CXCR4**	BV421	1/100	Biolegend	146511
**CXCR2**	PE	1/50	Biolegend	149304
**CD63**	Percp-Cy5.5	1/200	Biolegend	143912
**Siglec-F**	APCCy7	1/200	BD bioscience	565527

Appendix Table S3: Antibody panel for lung immunophenotyping

Target	Fluorophore	Dilution	Supplier	Catalogue Number
**Zombie NIR**	Zombie NIR	1/1000	Biolegend	423105
**CD45**	AF700	1/200	Biolegend	103128
**CD19**	Bv605	1/200	Biolegend	115540
**CD3**	PercpCy5.5	1/200	Biolegend	100328
**CD8**	FITC	1/200	Biolegend	100706
**CD4**	PeCy7	1/200	Biolegend	116016
**gdTCR**	PE	1/200	Biolegend	118108
**SiglecF**	AF647	1/200	BD Pharmigen	562680
**CD115**	Bv421	1/200	Biolegend	135513
**CD11b**	Bv650	1/200	Biolegend	101259
**Ly6G**	Bv711	1/200	Biolegend	127463
**F4/80**	Bv510	1/200	Biolegend	123135

Appendix Table S4: UPP1 expression is not upregulated in cancer associated fibroblasts in comparison to normal fibroblasts. UPP1 was assessed in publicly available proteomic datasets, and fold change in UPP1 expression was noted in cases where differential expression was identified when comparing UPP1 in cancer associated fibroblasts (CAFs) in comparison to normal fibroblasts (NFs) (Data Ref: Torres *et al*, 2013; Manousopoulou *et al*, 2018; Jaeschke *et al*, 2020).

PMID	Differential expression UPP1 CAF v NF
24025712	Not identified
29593339	Not differentially expressed
32984808	-2.49001

Appendix Table S5: Isotopically labelled internal standards for CD11b-DTR metabolomics

Standard	Supplier information
**^{13}C-labeled yeast extract**	(Cambridge Isotope Laboratory, Andover, MA, ISO1)
**$^{2}H_9$ choline**	(Cambridge Isotope Laboratory, Andover, MA, DLM-549)
**$^{13}C_4$ 3-hydroxybutyrate**	(Cambridge Isotope Laboratory, Andover, MA, CLM-3853)
**$^{13}C_6{}^{15}N_2$ cystine**	(Cambridge Isotope Laboratory, Andover, MA, CNLM4244)
**$^{13}C_3$ lactate**	(Sigma-Aldrich, Darmstadt, Germany, 485926)
**$^{13}C_6$ glucose**	(Cambridge Isotope Laboratory, Andover, MA, CLM-1396)
**$^{13}C_3$ serine**	(Cambridge Isotope Laboratory, Andover, MA, CLM-1574)
**$^{13}C_2$ glycine**	(Cambridge Isotope Laboratory, Andover, MA, CLM-1017)
**$^{13}C_5$ hypoxanthine**	(Cambridge Isotope Laboratory, Andover, MA, CLM8042)
**$^{13}C_2{}^{15}N$ taurine**	(Cambridge Isotope Laboratory, Andover, MA, CNLM-10253)
**$^{13}C_3$ glycerol**	(Cambridge Isotope Laboratory, Andover, MA, CLM-1510)
**$^{2}H_3$ creatinine**	(Cambridge Isotope Laboratory, Andover, MA, DLM-3653)

Appendix Table S6: Antibody panel for lymphoid characterisation

Target	Fluorophore	Dilution	Supplier	Catalogue Number
**zombie NIR**	Zombie NIR	1/1000	Biolegend	423105
**CD11b, CD11c, CD19**	BV605 (Dump)	1/200	Biolegend	101257, 117334, 115540
**PD-1**	APC	1/200	Biolegend	135210
**CD45**	A700	1/200	Biolegend	103128
**gdTCR**	PE	1/200	Biolegend	118108
**CD62L**	PE-Dazzle	1/200	Biolegend	104448
**CD4**	PE-Cy7	1/200	Biolegend	116016
**CD8**	PerCP-Cy5.5	1/200	Biolegend	100734
**CD44**	BUV395	1/200	BD Horizon	568507
**NKp46**	BV421	1/200	Biolegend	137611
**CD27**	BV510	1/200	Biolegend	124229
**CD69**	BV650	1/200	Biolegend	104541
**ICOS**	BV711	1/200	Biolegend	313548
**CD3**	BV785	1/200	Biolegend	100355

Appendix Table S7: Antibody panel for intracellular characterisation of T cell effector function

Target	Fluorophore	Dilution	Supplier	Catalogue Number
**zombie NIR**	Zombie NIR	1/1000	Biolegend	423105
**CD11b, CD11c, CD19**	BV605 (Dump)	1/200	Biolegend	101257, 117334, 115540
**granzyme B**	AF647	1/25	Biolegend	515406
**CD45**	A700	1/200	Biolegend	103128
**IL2**	PE	1/100	Biolegend	503808
**IFN gamma**	PE-Cy7	1/400	Life Tech (Invitrogen)	25-7311-82
**CD8**	PerCP-Cy5.5	1/200	Biolegend	100734
**TNF alpha**	BV711	1/25	Biolegend	506349
**CD3**	BV785	1/200	Biolegend	100355

Appendix Table S8: qRT-PCR primer sequences

Primer Name	Primer Sequence 5’ – 3’
**Actb F**	CATTGCTGACAGGATGCAGAAGG
**Actb R**	TGCTGGAAGGTGGACAGTGAGG
**Upp1 F**	GGAATCTTCGGTGTTTGCCACC
**Upp1 R**	GTGTTGATCTGGTCTCCTTGCAG
