# Supplementary figures and images for: Uridine phosphorylase-1 supports metastasis by altering immune and extracellular matrix landscapes
Source: EMBO Rep. 2025 Jul 23;26(17):4248–82. doi: 10.1038/s44319-025-00520-7 (PMC12420820; doi:10.1038/s44319-025-00520-7)

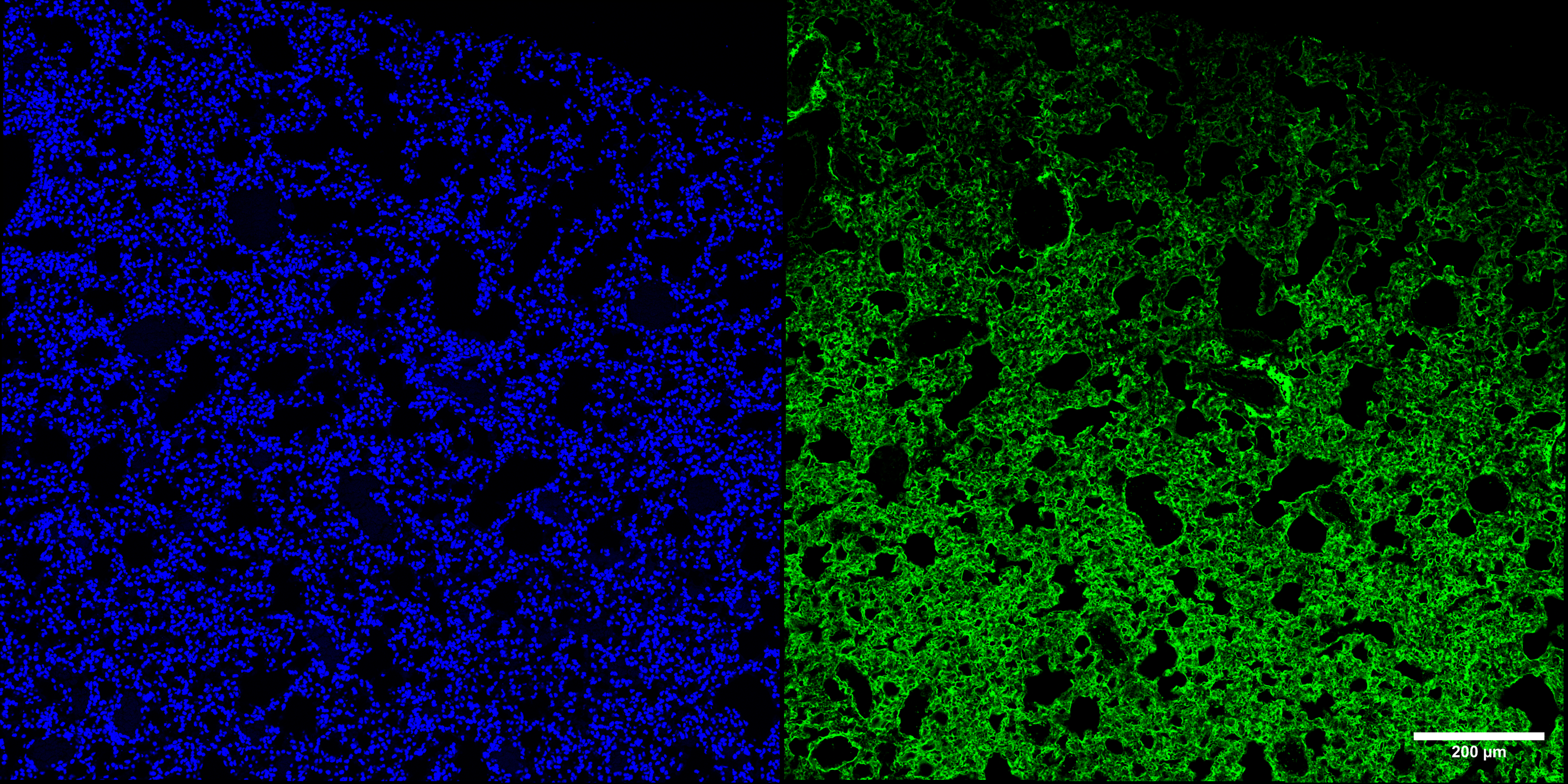

Supplement: Supplementary file 7 — Source data Fig. 5 [file 44319_2025_520_MOESM7_ESM.zip › EMBOR-2024-59902V3_Figure 5/EMBOR-2024-59902_Source Data Figure 5A Upp1++.tif]

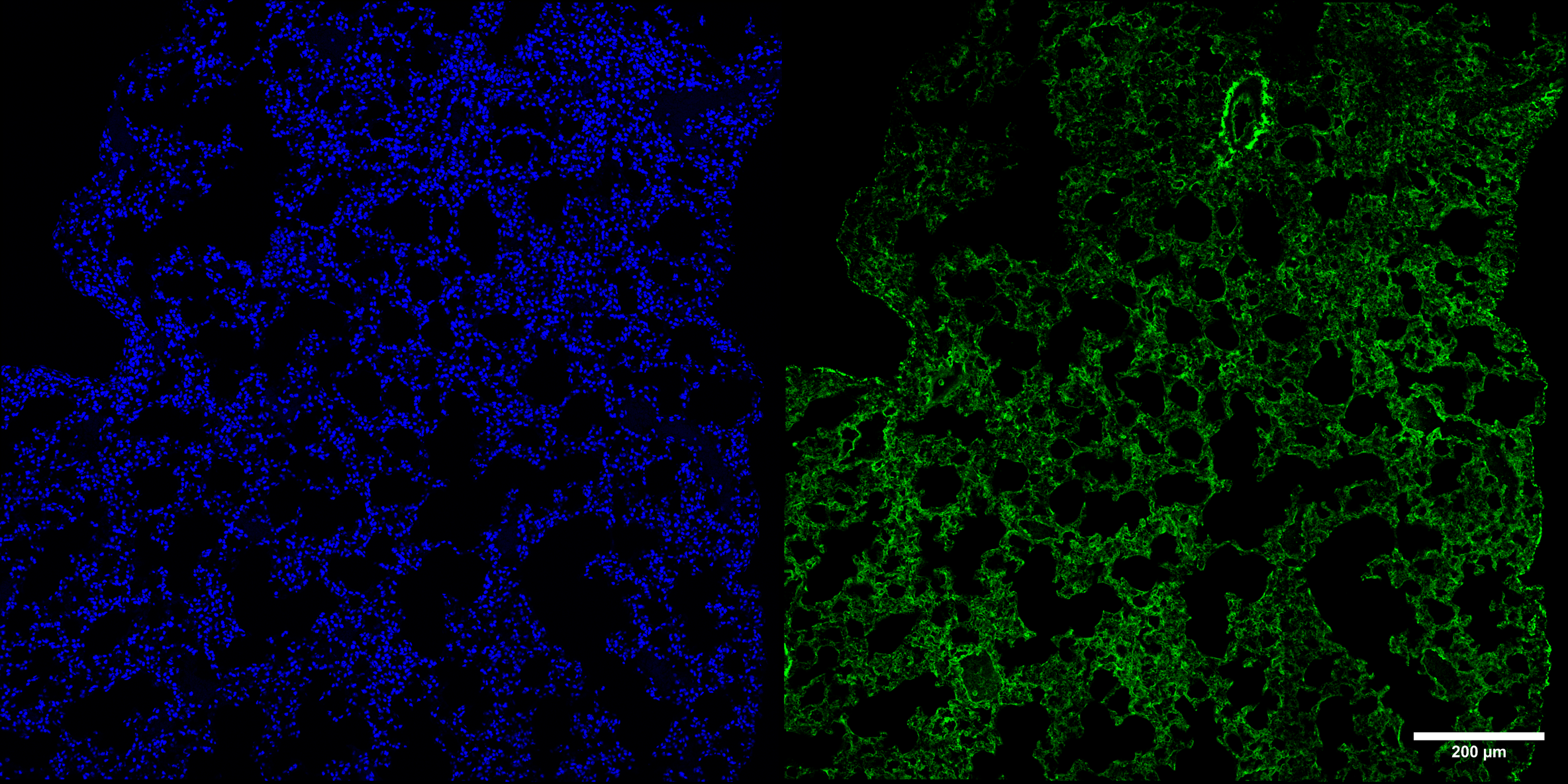

Supplement: Supplementary file 7 — Source data Fig. 5 [file 44319_2025_520_MOESM7_ESM.zip › EMBOR-2024-59902V3_Figure 5/EMBOR-2024-59902_Source Data Figure 5A Upp1--.tif]
