# Supplementary material for: Uridine phosphorylase-1 supports metastasis by altering immune and extracellular matrix landscapes
Source: EMBO Rep. 2025 Jul 23;26(17):4248–82. doi: 10.1038/s44319-025-00520-7 (PMC12420820; doi:10.1038/s44319-025-00520-7)
Supplement: Supplementary file 9 — Expanded View Figures [file 44319_2025_520_MOESM9_ESM.pdf]

Expanded View Figures

Figure EV1. Primary tumours, diet and age, are not responsible for the UPP1-dependent increases in uracil seen in metastatic cancer.

(**A**) Serum uracil was assessed by LC–MS in mice FVB/N ($n = 5$ mice), or mice that were transplanted with KP tumour fragments (tumours grown to 10 mm diameter, $n = 4$ *Upp1*$^{+/+}$ recipient mice, $n = 4$ *Upp1*$^{-/-}$ recipient mice). (**B**) *Upp1* was assessed in mammary gland ($n = 8$ mice), or *PyMT*$^{+}$ mammary tumour ($n = 31$ mice) by RNA-seq. (**C**) *Upp1* was assessed by qRT-PCR in *PyMT*$^{+}$ mammary tumours from mice that had 0 ($n = 7$ mice), 1–10 ($n = 16$ mice) or >10 ($n = 6$ mice) metastasis detectable by histological assessment of the lung. (**D**) *Upp1* in MMTV-*PyMT* tumours was assessed by qPCR and serum uracil in matched mice assessed by LC–MS ($n = 29$ mice in total, black dots represent mice with no metastasis, and pink and green dots represent mice with 1–10 or >10 metastases, respectively). (**E**) *Upp1* was assessed by RNA-seq from cell lines derived from primary mammary tumours (that were a consequence of fat-pad transplantation of *PyMT*$^{+}$ cell lines), or isogenic cells derived from micrometastases that formed in the lung following primary tumour resection (cell line generation and characterisation described in (Gounis et al, 2025) ($n = 6$ independent cell lines from $n = 6$ mice). (**F**, **G**) FVB/N mice were dosed daily, by oral gavage, with normal water or water containing 10 mM uracil for a total of 4 days. Mice were sacrificed 2 h post dosing on day 4. Uracil was then assessed in the serum (**F**), or kidney and lung (**G**) by LC–MS ($n = 4$ mice per experimental group). (**H**) Serum uracil in FVB/N mice younger and older than 90 days ($n = 3$ mice <90 days, and $n = 6$ mice >90 days old). (**I**) Age of mice at clinical endpoint presented in relation to the number of lung metastasis per mouse ($n = 30$ mice). Data Information: In bar graphs, data are presented as mean ± SEM. In (**A–D**, **F–I**) dots represent individual mice. In (**E**), each dot represents the average of technical triplicate repeats for each cell line. When more than 1 comparison is made (**A**, **C**, **G**) *P* values were calculated through one-way ANOVA, when 2 experimental groups were compared (**B**, **E**, **F**, **H**) unpaired *t* test is used, ns = not statistically significant. For correlation plots, Spearman Correlation statistics are presented.

 © The Author(s)

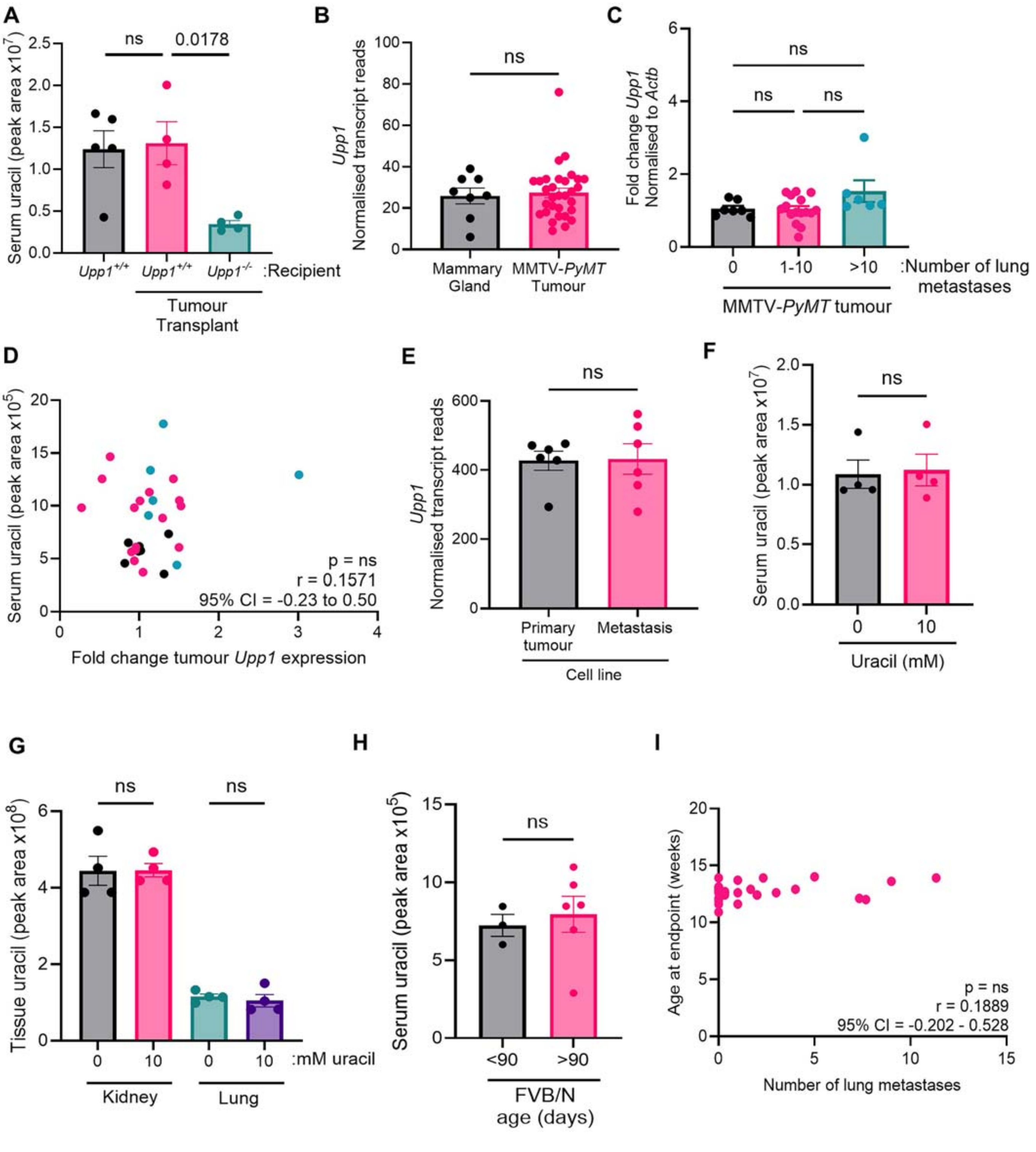
A
ns
0.0178
Serum uracil (peak area x10^7)
Upp1+/+ Upp1+/+ Upp1-/- :Recipient
Tumour Transplant
B
ns
Upp1 Normalised transcript reads
Mammary Gland
MMTV-PyMT Tumour
C
ns
ns
ns
Fold change Upp1 Normalised to Actb
0 1-10 >10 :Number of lung metastases
MMTV-PyMT tumour
D
Serum uracil (peak area x10^5)
p = ns
r = 0.1571
95% CI = -0.23 to 0.50
Fold change tumour Upp1 expression
E
ns
Upp1 Normalised transcript reads
Primary tumour
Metastasis
Cell line
F
ns
Serum uracil (peak area x10^7)
Uracil (mM)
G
ns
ns
Tissue uracil (peak area x10^8)
0 10 0 10 :mM uracil
Kidney
Lung
H
ns
Serum uracil (peak area x10^5)
<90 >90
FVB/N age (days)
I
Age at endpoint (weeks)
p = ns
r = 0.1889
95% CI = -0.202 - 0.528
Number of lung metastases

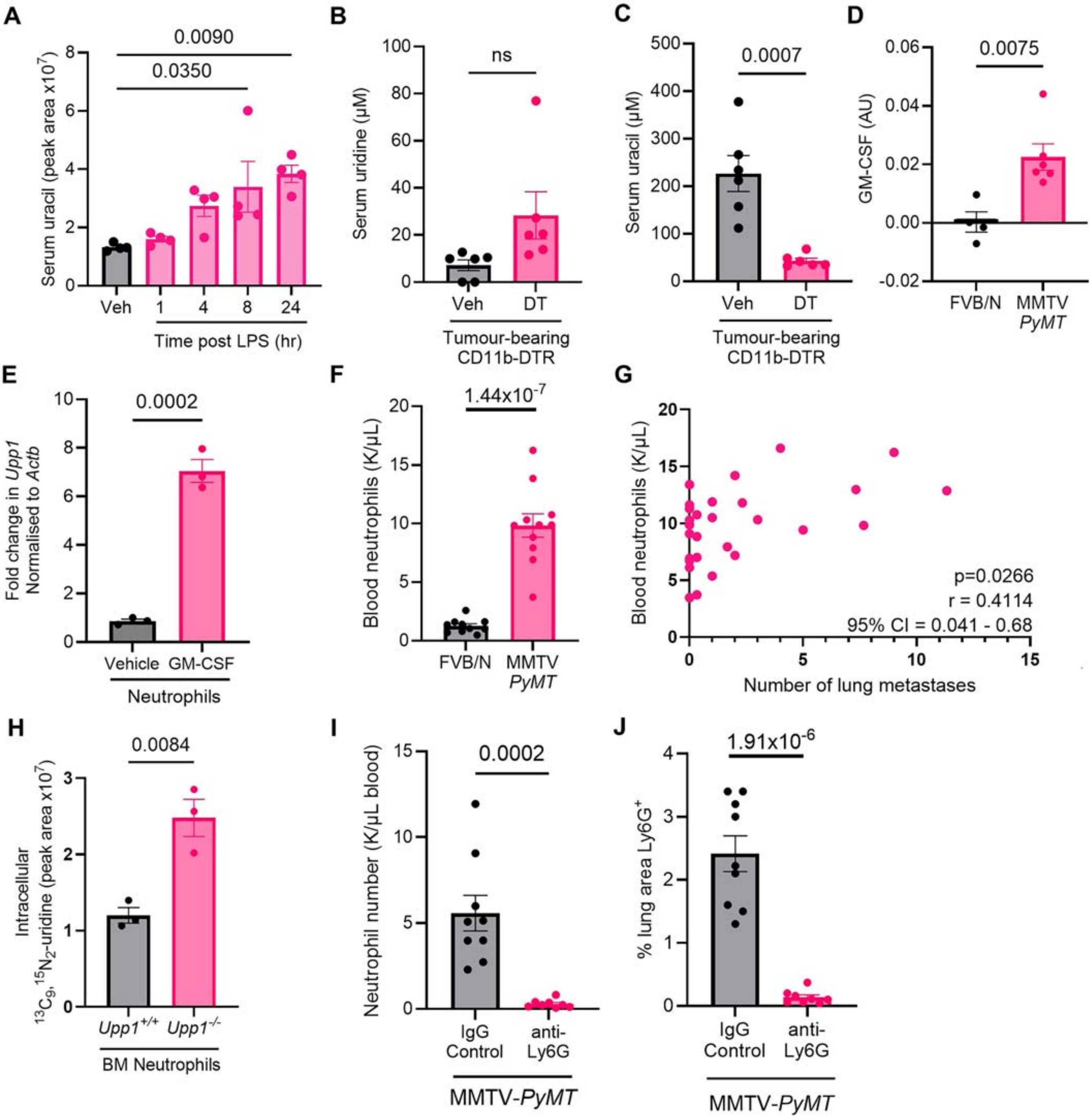
A
0.0090
0.0350
Serum uracil (peak area x10^7)
Veh 1 4 8 24
Time post LPS (hr)
B
ns
Serum uridine (μM)
Veh DT
Tumour-bearing CD11b-DTR
C
0.0007
Serum uracil (μM)
Veh DT
Tumour-bearing CD11b-DTR
D
0.0075
GM-CSF (AU)
FVB/N MMTV PyMT
E
0.0002
Fold change in Upp1 Normalised to Actb
Vehicle GM-CSF
Neutrophils
F
1.44x10^-7
Blood neutrophils (K/μL)
FVB/N MMTV PyMT
G
Blood neutrophils (K/μL)
p=0.0266
r = 0.4114
95% CI = 0.041 - 0.68
Number of lung metastases
H
0.0084
Intracellular 13C9, 15N2-uridine (peak area x10^7)
Upp1+/+ Upp1-/-
BM Neutrophils
I
0.0002
Neutrophil number (K/μL blood)
IgG Control anti-Ly6G
MMTV-PyMT
J
1.91x10^-6
% lung area Ly6G+
IgG Control anti-Ly6G
MMTV-PyMT

 © The Author(s)

Figure EV2. Neutrophils are the significant source of *Upp1* in metastatic mammary cancer.

(**A**) FVB/N mice were dosed intraperitoneal with 0.5 mg/kg lipopolysaccharide (LPS), or vehicle control, and serum uracil measured by LC–MS at defined timepoints post dosing ($n = 4$ mice per experimental group). (**B**, **C**) Mice bearing orthotopic PDAC tumours were treated with diphtheria toxin (DT) to ablate myeloid cells, and serum uridine (**B**) and uracil (**C**) were assessed after 24 h by LC–MS ($n = 6$ mice per experimental group). (**D**) GM-CSF was measured in the serum of FVB/N (n = 4 mice) and MMTV-*PyMT* tumour-bearing mice ($n = 6$ mice) by ELISA. (**E**) *Upp1* was assessed by qRT-PCR in BM neutrophils treated ex vivo with vehicle or 20 ng/mL GM-CSF for 24 h ($n = 3$ mice). (**F**) Blood neutrophils determined by IDEXX in MMTV-*PyMT* tumour-bearing mice ($n = 11$ mice) and 14-week-old FVB/N controls ($n = 10$ mice). (**G**) Number of blood neutrophils determined by IDEXX in MMTV-*PyMT* tumour-bearing mice at clinical endpoint ($n = 29$ mice), compared to number of lung metastases determined by histological analysis. (**H**) Intracellular $^{13}C_9,^{15}N_2$-uridine detected by LC–MS in BM neutrophils isolated from female FVB/N *Upp1*$^{+/+}$ and *Upp1*$^{-/-}$ mice, that were incubated for 24 h with $^{13}C_9,^{15}N_2$-uridine ($n = 3$ mice per experimental group). (**I**) Blood neutrophils determined by IDEXX and in MMTV-*PyMT* tumour-bearing mice treated with IgG control ($n = 9$ mice) or anti-Ly6G ($n = 8$ mice). (**J**) The proportion of lung area positive for Ly6G by immunohistochemistry for mice described in I (IgG control $n = 9$ mice; anti-Ly6G $n = 8$ mice). Data Information: In bar graphs dots represent individual mice and data are presented as mean ± SEM. When more than 1 comparison was made (**A**) *P* values were calculated through one-way ANOVA, when 2 experimental groups are compared (**B**–**F**, **H**–**J**) unpaired *t* test is used, ns = not statistically significant. For correlation plots (**G**), Spearman Correlation statistics are presented.

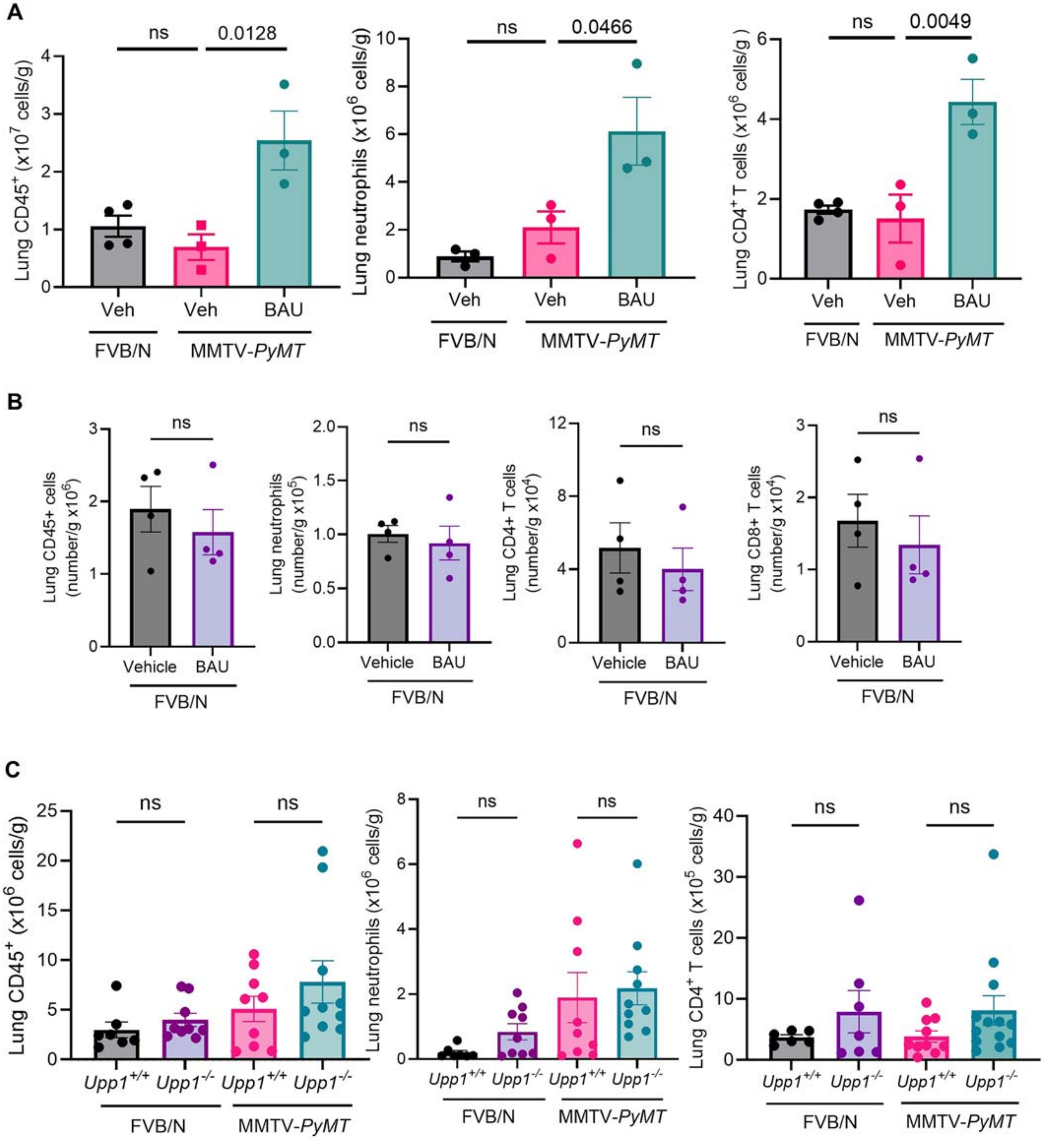

Figure EV3. The effect of UPP1 on the immune landscape of the lung.

Cell number per gram of lung for $CD45^+$, neutrophils, and $CD4^+$ T cells, assessed by flow cytometry, in the lungs of (**A**) MMTV-*PyMT* mice treated with vehicle ($n = 3$ mice) or BAU ($n = 3$ mice) from palpable tumour until mammary tumours reached 10–15 mm in diameter, and FVB/N ($n = 4$ mice) treated with vehicle for time-matched periods. (**B**) FVB/N mice treated with vehicle ($n = 4$ mice) or BAU ($n = 4$ mice) for 12 days. (**C**) MMTV-*PyMT*;*Upp1*$^{+/+}$ ($n = 9$ mice) and MMTV-*PyMT*;*Upp1*$^{-/-}$ ($n = 10$ mice) mammary tumour-bearing mice were harvested when one tumour measured 10–15 mm diameter. $N = 7$ and $n = 9$ FVB/N *Upp1*$^{+/+}$ and *Upp1*$^{-/-}$ mice were taken as age-matched controls, respectively. Data Information: In all cases, dots represent individual mice and data are presented as mean ± SEM. When more than 1 comparison is made (**A**, **C**) *P* values are calculated through one-way ANOVA, when 2 experimental groups are compared (**B**) unpaired *t* test is used, ns = not statistically significant.

 © The Author(s)

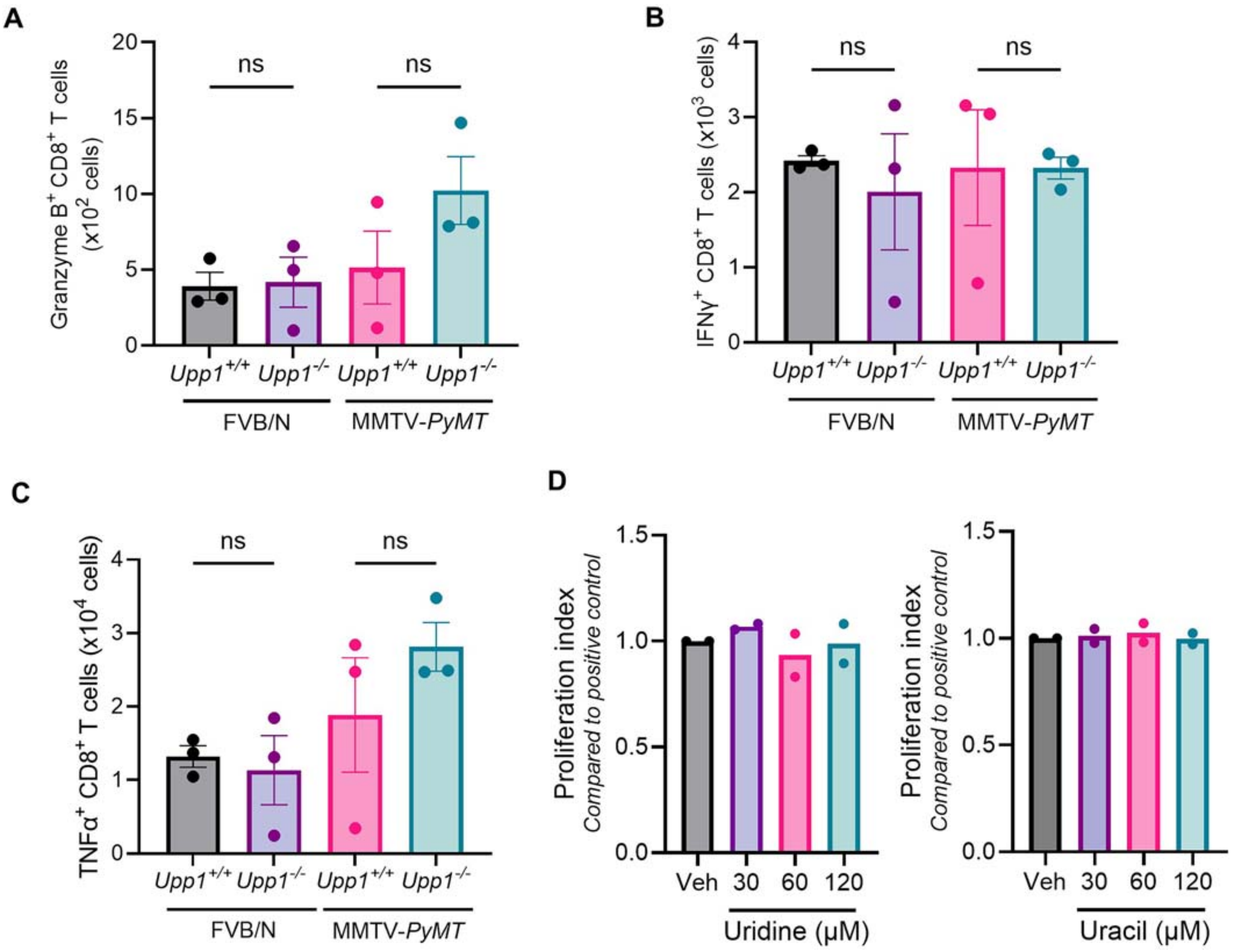

Figure EV4. Understanding T-cell effector function in the absence of *Upp1*.

(**A–C**) Lungs of MMTV-*PyMT*;*Upp1*$^{+/+}$ and MMTV-*PyMT*;*Upp1*$^{-/-}$ tumour-bearing mice were harvested when one tumour measured 10–15 mm diameter. FVB/N age-matched controls were taken in parallel. Cells were prepared as described in the methods for intracellular T-cell staining ($n = 3$ mice per experimental group). (**D**) T cells were isolated from FVB/N mice, incubated in culture with CD3/CD28 dynabeads to stimulate proliferation in medium containing 30, 60 and 120 µM uridine or uracil. Proliferation index was calculated compared to the positive control of T cells and vehicle alone stimulated with beads ($n = 2$ mice). Data Information: In all cases, dots represent individual mice. (**A–C**) Data are mean ± SEM, and *P* values were calculated by one-way ANOVA. As data in (**D**) is $n = 2$, data are presented as mean with datapoints, without error bars and statistics.

© The Author(s)

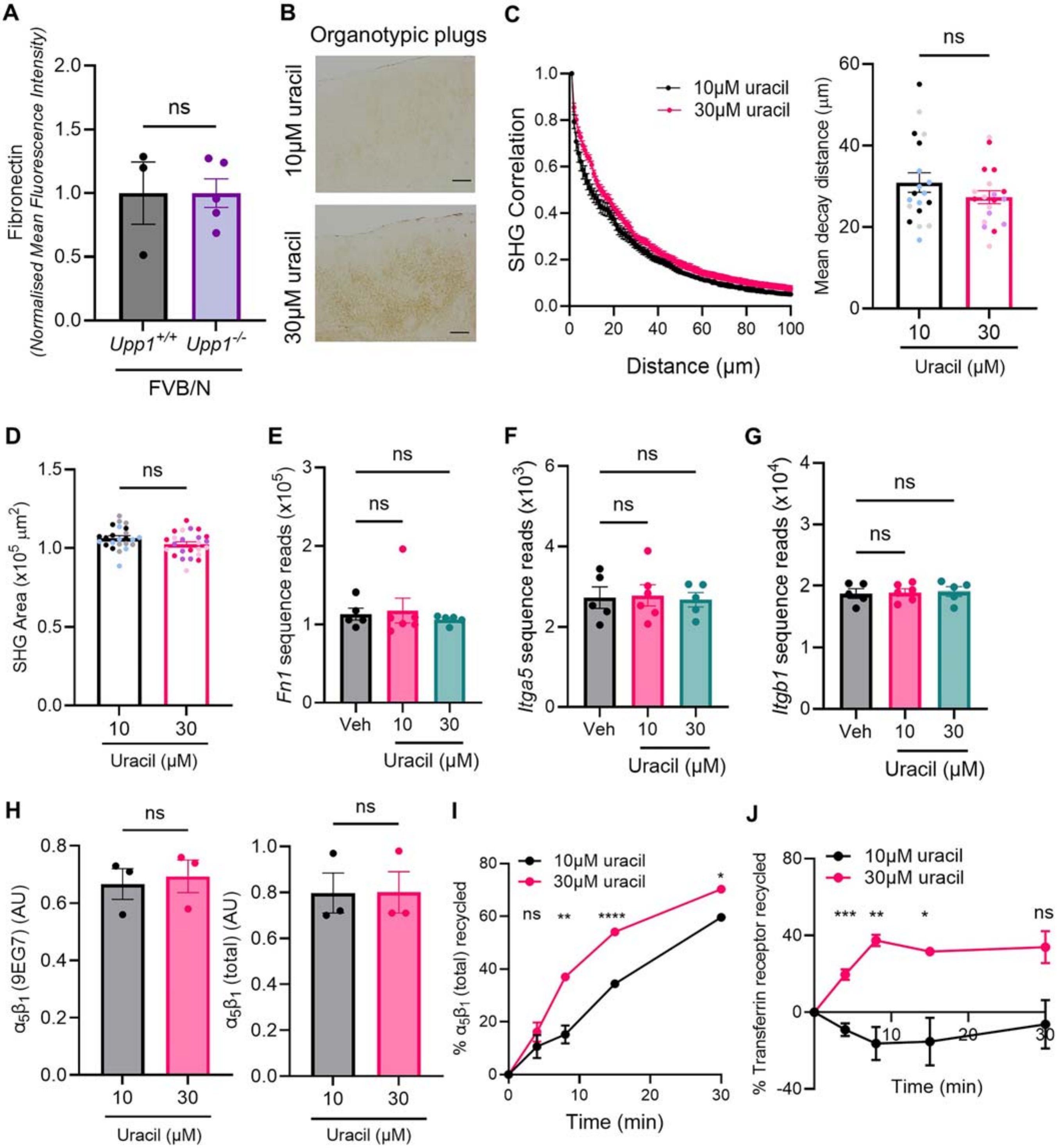
A
Fibronectin (Normalised Mean Fluorescence Intensity)
ns
Upp1+/+ Upp1-/-
FVB/N
B
Organotypic plugs
10μM uracil
30μM uracil
C
SHG Correlation
10μM uracil
30μM uracil
Distance (μm)
ns
Mean decay distance (μm)
10 30
Uracil (μM)
D
SHG Area (x10^5 μm^2)
ns
Uracil (μM)
E
Fn1 sequence reads (x10^5)
ns
Veh 10 30
Uracil (μM)
F
Itga5 sequence reads (x10^3)
ns
G
Itgb1 sequence reads (x10^4)
ns
H
α5β1 (9EG7) (AU)
α5β1 (total) (AU)
ns
I
% α5β1 (total) recycled
10μM uracil
30μM uracil
ns ** **** *
Time (min)
J
% Transferrin receptor recycled
*** ** * ns
Time (min)

 © The Author(s)

Figure EV5. Understanding the effect of *Upp1* and uracil on ECM deposition.

(**A**) Lung fibronectin was assessed in FVB/N *Upp1*$^{+/+}$ ($n = 3$ mice) and *Upp1*$^{-/-}$ ($n = 5$ mice) mice by immunofluorescence, age-matched to those presented in Fig. 5A, B. (**B**) Fibroblasts were added to rat tail collagen with medium containing 10 µM or 30 µM uracil to make organotypic plugs. Plugs were contracted by fibroblasts for 7 days and stained for fibronectin by immunohistochemistry. Representative images are shown for $n = 4$ biological repeat experiments, scale bar 100 µm. (**C**, **D**) Fibrillar collagen was imaged by second harmonic generation (SHG) microscopy in organotypic plugs contracted by fibroblasts supplemented with 10 or 30 µM uracil. A threshold was applied to the SHG signal and the area of SHG coverage per field of view was determined. The organisation of fibrillar collagen in each field of view was assessed by applying grey level co-occurrence matrix. The mean correlation decay curves from each experimental condition, and the mean of the decay distances derived from those curves, are presented (n = 3 biological repeat experiments colour coded). (**E–G**) RNA-Seq transcript reads from fibroblasts treated with vehicle, 10 µM and 30 µM uracil for 24 h, for fibronectin (*Fn1*), integrin α_5 (*Itga5*) and integrin β_1 (*Itgb1*) ($n = 5$ biological repeats for vehicle and 30 µM condition, $n = 6$ biological repeats for 10 µM uracil). (**H**) Total protein levels quantified from total cell lysate via ELISA, arbitrary units (AU) ($n = 3$ biological repeats). (**I**) Recycling of the total levels of the fibronectin receptor, $\alpha_5\beta_1$ integrin, assessed in fibroblasts treated with 10 µM or 30 µM uracil for 24 h ($n = 3$ biological repeats). (**J**) Recycling of transferrin receptor assessed in fibroblasts treated with 10 µM or 30 µM uracil for 24 h ($n = 3$ biological repeats). Data Information: In all graphs, data are mean ± SEM. In (**A**) dots represent individual mice. In (**C**, **D**), dots are $n = 24$ fields of view across $n = 3$ colour coded biological repeat experiments. $N = 19$ fields of view are represented for mean decay distance as 6 fields of view did not conform to 2 phase decay curves and so could not be fitted in the equation. In (**E–J**), dots represent biological experiment repeats. When 2 experimental groups are compared (**A**, **C**, **D**, **H–J**) unpaired *t* test is used. For (**I**), $^*P = 0.0042$; $^{**}P = 0.0041$, $^{****}P = 4.62 \times 10^{-5}$. For (**J**), $^*P = 0.0207$; $^{**}P = 0.0042$; $^{***}P = 0.0025$. When more than 1 comparison is made (**E–G**) *P* values are calculated through one-way ANOVA, ns = not statistically significant.

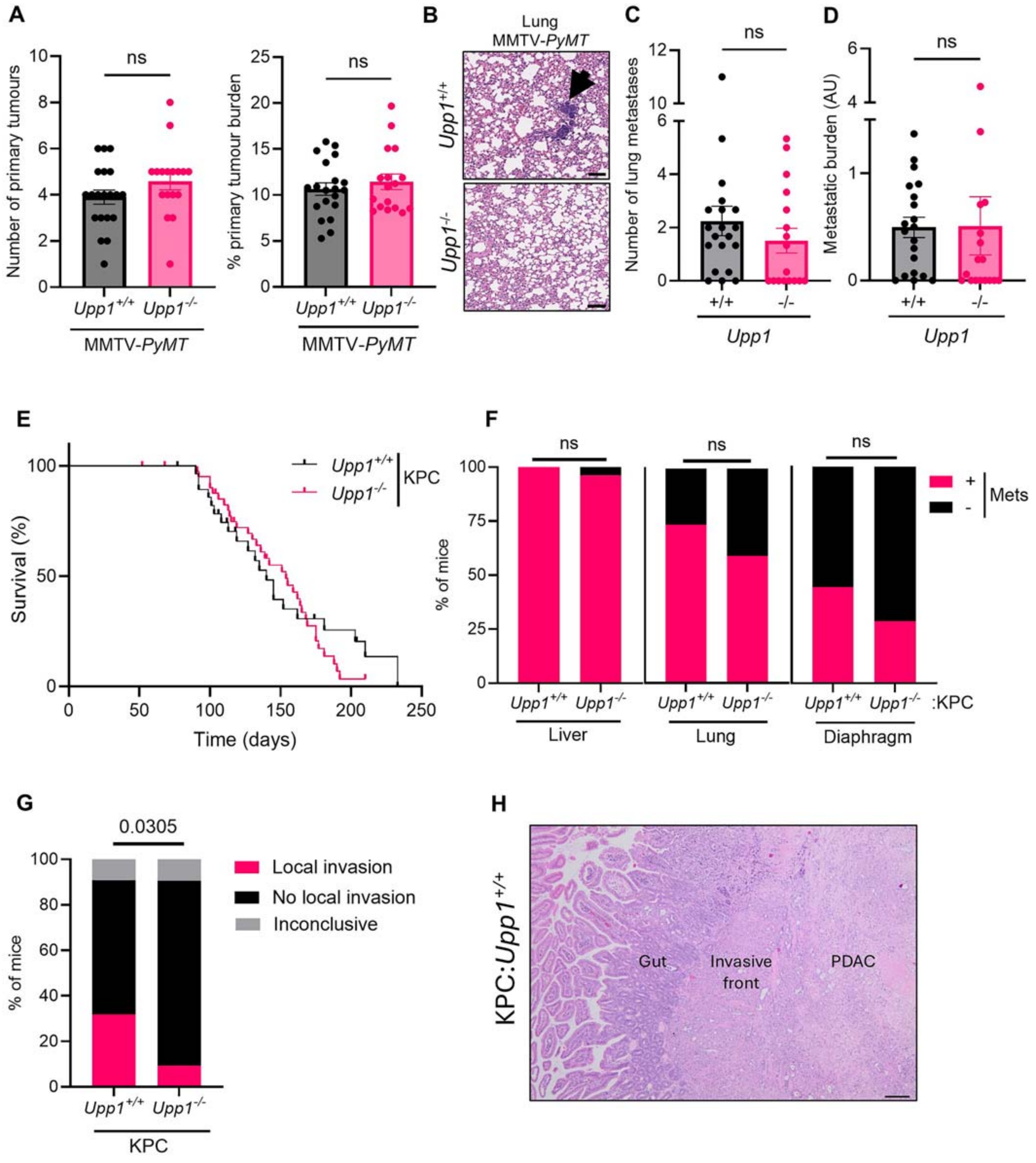
A
Number of primary tumours
% primary tumour burden
ns
Upp1+/+ Upp1-/-
MMTV-PyMT
B
Lung
MMTV-PyMT
Upp1+/+
Upp1-/-
C
Number of lung metastases
D
Metastatic burden (AU)
+/+ -/-
Upp1
E
Survival (%)
Time (days)
KPC
F
% of mice
Mets
Liver
Lung
Diaphragm
:KPC
G
0.0305
Local invasion
No local invasion
Inconclusive
KPC
H
KPC:Upp1+/+
Gut
Invasive front
PDAC

Figure EV6. Understanding the effect of *Upp1* loss in genetically engineered mouse models of metastatic cancer.

(**A**) Mammary tumour number and burden in mice MMTV-*PyMT*;*Upp1*$^{+/+}$ ($n = 20$ mice) and MMTV-*PyMT*;*Upp1*$^{-/-}$ ($n = 17$ mice) at clinical endpoint. (**B**) Representative H&E stains for the lungs of MMTV-*PyMT*;*Upp1*$^{+/+}$ and MMTV-*PyMT*;*Upp1*$^{-/-}$ mice described in (**A**), scale bar 100 µm. Arrow highlights a metastatic lesion. (**C**, **D**) Average number of lung metastases (**C**) and average lung metastatic burden (**D**), determined by histological H&E assessment of serial lung sections from MMTV-*PyMT*;*Upp1*$^{+/+}$ ($n = 20$ mice) and MMTV-*PyMT*;*Upp1*$^{-/-}$ ($n = 17$ mice). (**E**) Overall survival of KPC:*Upp1*$^{+/+}$ ($n = 22$ mice) and KPC:*Upp1*$^{-/-}$ mice ($n = 33$ mice). Mice that were sacrificed due to indications other than PDAC were censored (amounting to $n = 7$ *Upp1*$^{+/+}$ mice and $n = 10$ *Upp1*$^{-/-}$ mice). (**F**) The proportion of mice with metastasis detected by histological H&E assessment of serial sections of liver and lung, KPC:*Upp1*$^{+/+}$ ($n = 19$ mice) and KPC:*Upp1*$^{-/-}$ ($n = 27$ mice), and the proportion of mice with microscopic diaphragm metastasis for KPC:*Upp1*$^{+/+}$ ($n = 18$ mice) and KPC:*Upp1*$^{-/-}$ mice ($n = 21$ mice). (**G**) Local invasion was determined for KPC:*Upp1*$^{+/+}$ ($n = 22$) and KPC:*Upp1*$^{-/-}$ ($n = 32$) mice. Inconclusive annotation refers to samples that were unclear at necropsy as to whether full invasion had occurred. (**H**) Representative image of local invasion that was scored at necropsy, namely the attachment and invasion of the primary PDAC to other organs within the abdominal cavity. Example shown is from a KPC:*Upp1*$^{+/+}$ mouse, with PDAC invading intestinal tissue, scale bar 200 µm. Data Information: In (**A**, **C**, **D**) dots represent individual mice, data are presented as mean ± SEM, and P values were calculated by unpaired t test, ns = not statistically significant. In (**E**), log-rank (Mantel–Cox) test $P = 0.5926$. In (**F**, **G**), P values calculated by chi-squared test.

© The Author(s)
